# Aboriginal and Torres Strait Islander Dental Program Evaluations: A Mixed‐Methods Systematic Review

**DOI:** 10.1111/cdoe.70023

**Published:** 2025-08-27

**Authors:** Alexander Pham, Joanne Hedges, Emma Flanagan, Tiyanna Mastrosavas, Lisa Jamieson, Sonia Nath

**Affiliations:** ^1^ Adelaide Dental School The University of Adelaide Adelaide South Australia Australia; ^2^ School of Public Health The University of Adelaide Adelaide South Australia Australia

**Keywords:** aboriginal, dentistry, first nations, health program evaluation, oral health, public health dentistry, Torres Strait Islander

## Abstract

**Objective:**

Over the last 20 years, the disparity in oral health between Aboriginal and Torres Strait Islander Australians and other Australians has continued to grow. This suggests that further programmes and programme improvements are needed to reach equitable oral health outcomes for Aboriginal and Torres Strait Islander Peoples. This mixed methods systematic review aims to assess Aboriginal and Torres Strait Islander Dental Programme Evaluations by measuring outcomes and cultural safety via the Lowitja Institute Evaluation Framework to Improve Aboriginal and Torres Strait Islander Health.

**Methods:**

Databases searched were PubMed, Embase, Dentistry & Oral Sciences Source, Public Health Database and Scopus. All years were included. The date of the last search was the 1 May 2025. Published articles researching dental health programmes with Aboriginal and Torres Strait Islander participants in Australia were identified. Quantitative, qualitative and mixed methods studies were included. The Joanna Briggs Institute approach to Mixed‐Methods Systematic Reviews was followed, except for a deviation in critical appraisal, which utilised the Quality Assessment for Diverse Studies (QuADs) instead. This review protocol was registered in PROSPERO (CRD42025637868) a priori.

**Results:**

There were 54 studies included. New South Wales, South Australia, Queensland, Northern Territory and Western Australia were the states with the most data. The research designs included qualitative, quantitative and mixed‐methods approaches. Most studies were of relatively high quality, as assessed by the QuADS criteria. Evaluations of dental programs for Aboriginal and Torres Strait Islander communities largely adhered to the Lowitja Framework, particularly in shared responsibility, partnerships and active engagement with Aboriginal and Torres Strait Islander peoples and their communities. Program effectiveness was primarily assessed through reductions in dental decay and participant feedback. Findings may be limited because the Lowitja Framework was developed via evaluation tenders, and the studies included were sourced from research articles.

**Conclusions:**

Strong partnerships between programs, evaluation teams and Aboriginal and Torres Strait Islander communities are critical for cultural safety. Effective study designs should be used, and culturally relevant and holistic outcome measures should be chosen. Lessons learnt from this systematic review can be used to improve the effectiveness of Aboriginal and Torres Strait Islander dental programme evaluations.

## 1 Introduction

In the 2022 Global Oral Health Report, the World Health Organisation (WHO) states that oral health inequalities are caused by ‘complex…country‐specific historical, economic, cultural and social or political factors’ [1](p. 15), and this is compounded for Indigenous peoples across the world [1]. Aboriginal and Torres Strait Islander Australians experience worse oral health than other Australians leading to pain, hospitalisations and decreased quality of life [2]. This is worsened by the lack of cultural safety, affordability and accessibility of dental services [3]. The dental health gap between Aboriginal and Torres Strait Islander Australians and other Australians has grown over the last 20 years [4]. Worsening trends have also been reported in Canada, New Zealand and Latin American countries, with key drivers being systemic racism and colonial legacies, social disadvantage and limited Indigenous representation in the oral health workforce [5]. One positive driver of change could be better dental programs for Aboriginal and Torres Strait Islander Peoples [6].

Health program evaluations maximise benefits by measuring effectiveness, identifying weaknesses and guiding improvements [7]. Evaluations of Aboriginal and Torres Strait Islander health programs have lacked quality and comprehensiveness [8], which leads to: ‘…the inappropriate funding of projects and programs that don't work at the expense of those that do’ [9] (p. 10). This has been due to a focus on clinical markers of success, and a lack of cultural relevancy of measured outcomes [10]. To address this, the Lowitja Institute developed an Evaluation Framework to Improve Aboriginal and Torres Strait Islander Health [10], valuing success by holistic improvements in health, cultural safety and co‐design. The Lowitja Framework was developed by Aboriginal and Torres Strait Islander researchers who analysed health evaluations from peer reviewed literature and evaluation tenders. This framework was released in 2018 and has not had widespread uptake. This may be due to health programs lacking funding models responsive to community needs and instead over‐emphasising clinical outcomes [11].

Similar literature reviews include Lokuge et al.'s systematic review of Aboriginal and Torres Strait Islander health program evaluations in Australia [8] and Maddox et al.'s review of health program evaluations for Indigenous Peoples in Australia, Canada, New Zealand and the United States [12]. Similar dental health‐related review performed or planned include Patel et al.'s scoping review [6] and Do et al.'s scoping review [13]. Patel et al.'s scoping review found that partnerships between community and health researchers and organisations showed deeper understanding of community needs; although programs were at risk of failure if interventions did not target socio‐economical determinants of health [6]. Do et al.'s scoping review described the settings, workforce and interventions involved in Aboriginal and Torres Strait Islander dental health [13]. These reviews provided details of interventions used in Aboriginal and Torres Strait Islander dental programs, however did not analyse the evaluation method. This study is unique in its focus on dental health, evaluations and cultural safety and its use of the Lowitja Framework, which was developed with Aboriginal and Torres Strait Islander Peoples of Australia. Culturally safe practice involves the acknowledgement of colonisation and systemic racism, individual racism, recognition of the importance of self‐determined decision making, and a safe working environment for Aboriginal and Torres Strait Islander people [14].

The aim of this study was to assess the evaluations of Aboriginal and Torres Strait Islander dental programs. The primary research question in PICo (Population, Interest, Context) format is: ‘In Aboriginal and Torres Strait Islander adults and children (P), how are dental health programs evaluated (I) via the Lowitja Framework (C) in terms of their cultural safety and strength of partnerships?’ It was hypothesised that strong partnerships between the evaluation team and Aboriginal and Torres Strait Islander communities will improve the cultural safety and effectiveness of dental programs.

## 2 Method

Aboriginal and Torres Strait Islander dental programs have been evaluated quantitatively and qualitatively by previous researchers. Therefore, a mixed methods approach was used to capture quantitative outcomes and lived experiences, cultural safety and implementation challenges. This approach ensures that no single methodology is excluded and offers a richer and more holistic understanding of program effectiveness and context [15]. This review used the Joanna Briggs Institute (JBI) convergent mixed methods systematic review methodology [15]. The PRISMA checklist was used when writing the report [16] (Tables S1 and S2). This review protocol was registered in PROSPERO (CRD42025637868) a priori.

### 2.1 Eligibility Criteria

To ensure a comprehensive and systematic approach to our literature search and study selection, we used the Sample, Phenomenon of Interest, Design, Evaluation and Research type (SPIDER) framework [17] (Table S3). This review included studies of Aboriginal and Torres Strait Islander Peoples (Sample), in dental health programmes (Phenomenon of interest), with study designs including qualitative, quantitative and mixed methods methodologies (Design and Research type), evaluating perspectives, views, preferences, experiences, effectiveness and cultural safety (Evaluation). There were no restrictions on age or gender, and only studies in English were included. The quantitative studies used experimental methods such as randomised controlled trials (RCTs) and quasi‐experimental studies, as well as observational studies such as cohort studies, case–control studies and cross‐sectional surveys. The qualitative studies used methods such as one‐on‐one interviews, focus groups, clinical yarning and case studies. Mixed methods studies combining quantitative and qualitative approaches were also included. The review included primary studies but also considered secondary studies that provided further analysis of primary data. Studies were undertaken in dental clinics, Aboriginal Community‐Controlled Health Services and community settings.

Articles were excluded if they were systematic and scoping reviews or study protocols. Studies were excluded if the evaluated programmes were not developed for Aboriginal and Torres Strait Islander Peoples. These excluded studies lacked information regarding partnerships, engagement and capacity building in Aboriginal and Torres Strait Islander Communities.

### 2.2 Evaluation Framework

The main outcome of this study was the evaluation of dental health programs for Aboriginal and Torres Strait Islander peoples using the Lowitja Institute's Evaluation Framework to Improve Aboriginal and Torres Strait Islander Health [10]. This consists of 11 criteria including partnerships with Aboriginal and Torres Strait Islander organisations and communities; shared responsibility; community engagement; capacity building; equity; accountability; evidence‐based practices; holistic health concepts; cultural competence; data governance and intellectual property; and capitalising on Indigenous strengths.

### 2.3 Search Strategy and Database Selection

The search aimed to identify all Aboriginal and Torres Strait Islander dental programs published in scientific literature and consisted of two key concepts: ‘Aboriginal and Torres Strait Islander People’ and ‘Dental Care’. The databases searched were PubMed, Embase, Dentistry & Oral Sciences Source, Public Health Database and Scopus. Databases were chosen, and logic grids were developed with the University of Adelaide Liaison Librarians. Keywords were searched in titles and abstracts (logic grids for databases available in Table S4). Grey literature was excluded due to feasibility constraints and the lack of centralised databases for Aboriginal and Torres Strait Islander oral health program evaluations. Identifying relevant literature would require manual searching through each Aboriginal Community Controlled Health Organisation website as well as each state government dental service platforms which do not routinely publish program evaluations [18]. The database search was conducted from 1 October 2024 to 1 May 2025, and references were compiled into EndNote Version 21 [19].

### 2.4 Study Selection

References were imported into Covidence [20], and duplicates were automatically identified and removed. Two independent researchers (AP & SN) conducted a pilot test of the title and abstract screening process using a sample of 15 studies. Once in agreement on criteria and methods, the reviewers proceeded with the full title and abstract screening. Two researchers (AP & SN) independently conducted the full‐text review. Reasons for excluding full‐text studies were recorded. Disagreements at any stage of the selection process were resolved through discussion with a third reviewer (LMJ).

### 2.5 Assessment of Methodological Quality

The included studies were critically appraised using the Quality Assessment with Diverse Studies (QuADS) [21]. The QuADS utilises a unified framework, allowing consistent appraisal across all studies which allows new findings to be synthesised from a range of designs instead of comparing and ranking clinical effectiveness of interventions. This review is exploratory and integrative and unlike individual appraisal tools, the QuADS does not impose rigid thresholds for quality appraisal, which is especially beneficial where studies are less rigorous although contextually rich. This was a more streamlined and comparable approach compared to the JBI method (using individual critical appraisal tools for each study type) [15]. Pilot testing of critical appraisal was done for five studies, followed by full appraisal (AP & SN).

### 2.6 Data Extraction, Transformation, Synthesis, Integration and Analysis

The chosen articles had data extracted using a custom data collection form inspired by the SPIDER framework. Data extracted included the type of study, data source type, sample size, description of study participants, intervention and comparator, study setting and quantitative outcomes.

A convergent‐integrated approach for mixed methods systematic reviews [15, 22] was utilised to compare quantitative and qualitative data. Quantitative data was ‘qualitized’ into qualitative data before analysis. After quantitative outcomes were extracted, they were converted to qualitative data in relation to the relevant Lowitja Criteria for example, in answer to the ‘Evidence‐Based’ criteria, a reason for meeting the criteria was the decrease in caries showing that the evaluation was evidence‐based. Additionally, all other qualitative data answering the Lowitja Criteria were extracted. The criterion was recorded as ‘unclear’ if it was not met. Data extraction was completed independently by two reviewers (AP & SN). The criteria for ‘Shared Responsibility’ and ‘Partnerships with Aboriginal and Torres Strait Islander Communities & Organisations’ was merged into ‘Shared Responsibility’ because the indicators were identical [10].

Based on the data extraction table, themes were developed with regard to which criteria were more often met compared to which criteria were commonly not met. Based on the data extraction table, another table was created to measure evaluated outcomes (e.g., caries or qualitative perspectives), adult or child, location of setting and study design. The findings are presented below in the discussion and in table format.

## 3 Results

### 3.1 Study Inclusion

**FIGURE 1:**
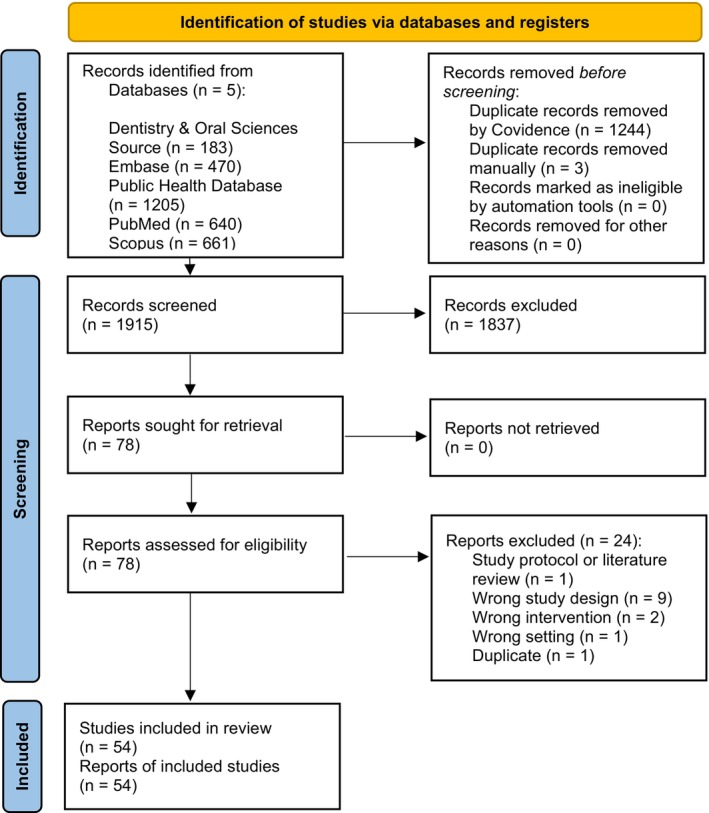
Preferred Reporting Items for Systematic Reviews and Meta‐Analyses (PRISMA) flow chart [12].

Study inclusion is detailed in the PRISMA diagram in Figure 1. There were 3159 studies identified from database searches, and 1241 references were identified as duplicates and removed by Covidence. Three were identified by the research team as duplicates and removed. The remaining 1915 studies were screened for title and abstract based on inclusion and exclusion criteria, with 1837 records excluded and 78 studies sought full‐text review. Twenty‐four studies were then excluded due to incorrect settings, interventions, designs and study protocols (description in PRISMA diagram in Figure 1 and details in Table S5). Fifty‐four studies were included in this systematic review.

## 4 Findings

### 4.1 Methodological Quality

Many studies demonstrated higher relative quality based on the QuADS assessment (Table S6, Figure S1). Lower‐quality studies were predominantly case reports with poorly defined aims. The weakest QuADS criteria was ‘appropriate sampling to address the research aim’ and ‘provision of recruitment data’. Quantitative studies often did not estimate the required sample size needed to achieve significant outcomes prior to participant recruitment or report reasons for non‐participation, which limits the statistical power and generalizability of the findings. In qualitative studies, while some mentioned aiming for data saturation, most did not explain the rationale for their sampling strategy or describe how saturation was assessed. Instead, participant recruitment may have been limited by convenience or practical constraints, without enough discussion about how sample size or selection affected the transferability of the findings. These limitations highlight important areas for improvement in future research.

### 4.2 Characteristics of Included Studies

The included studies and their characteristics are available in Table S7. Studies were conducted in the States and Territories of Australia, including New South Wales (16) [23, 24, 25, 26, 27, 28, 29, 30, 31, 32, 33, 34, 35, 36, 37, 38], South Australia (14) [39, 40, 41, 42, 43, 44, 45, 46, 47, 48, 49, 50, 51, 52] Queensland (10) [46, 53, 54, 55, 56, 57, 58, 59, 60, 61], Northern Territory (9) [46, 62, 63, 64, 65, 66, 67, 68, 69], Western Australia (8) [46, 70, 71, 72, 73, 74, 75, 76], and Tasmania (1) [46]. Studies from the Australian Capital Territory and Victoria were not present. One study used data from 5 states and territories [46]. Thirty‐nine studies specified rural areas [24, 25, 26, 27, 28, 29, 30, 34, 35, 42, 43, 47, 48, 49, 50, 53, 54, 55, 56, 57, 58, 59, 60, 61, 62, 63, 64, 65, 66, 67, 68, 69, 70, 71, 72, 73, 74, 75, 76] and 15 were unspecified and may have been based in metropolitan or a mix of metropolitan and rural areas [23, 31, 32, 33, 36, 37, 38, 39, 40, 41, 44, 45, 46, 51, 52]. Study designs included narrative/case studies (11) [25, 28, 39, 45, 47, 48, 49, 58, 68, 73, 75], RCTs (9) [40, 41, 43, 44, 51, 52, 63, 65, 66], qualitative (8) [27, 31, 33, 42, 57, 61, 74, 76], and quasi‐experimental (6) [37, 55, 56, 60, 62, 69]. Less common study designs were cluster randomised trials [34, 50, 70, 71], mixed methods [23, 35, 36], retrospective [29, 46, 59, 72], cross‐sectional [24, 32], longitudinal [53, 54], survey [38], cohort [26, 30], and community randomised trials [67]. Twenty‐nine studies were child‐focused [25, 26, 27, 30, 34, 35, 37, 39, 40, 41, 44, 46, 47, 49, 50, 51, 52, 53, 54, 55, 56, 59, 60, 62, 63, 67, 70, 71, 72], 13 were on adults and children [23, 24, 28, 29, 45, 48, 58, 64, 68, 69, 73, 75, 76], and 12 on adults [31, 32, 33, 36, 38, 42, 43, 57, 61, 65, 66, 74]. Some child‐focused studies included the perspective of adults (e.g., parents or healthcare workers) and were categorised as adult‐focused. The publishing years of these studies ranged from 2005 [48] to 2024 [45].

### 4.3 Outcomes Measured

#### 4.3.1 Caries, Participant Feedback, Narrative Recounts of Programs

The outcomes measured and their frequencies are presented in Table 1. The most common outcomes were caries or Decayed, Missing, Filled, or Treated indices (DMFT) (17) [26, 34, 37, 40, 41, 44, 46, 47, 50, 53, 54, 56, 60, 62, 63, 67, 71], and participant feedback (16) [23, 24, 27, 30, 31, 32, 33, 36, 38, 42, 45, 57, 61, 68, 74, 76]. Caries and DMFT were assessed through epidemiological or clinical studies. Study designs included RCTs or variations such as community or cluster randomisation trials.

**TABLE 1:** Outcomes measured and related studies.

Outcome	No. of studies	Reference no. of studies
Caries or DMFT	17	[19, 27, 30, 33, 34, 37, 39, 40, 43, 46, 47, 49, 53, 55, 56, 60, 64]
Participant Feedback	16	[16, 17, 20, 23, 24, 25, 26, 29, 31, 35, 38, 50, 54, 61, 67, 69]
Narrative Recounts of Programs	7	[28, 32, 41, 42, 54, 57, 68]
Process Evaluation	7	[18, 21, 28, 43, 52, 65, 66]
Health Economic Evaluation	5	[22, 48, 51, 57, 62]
Other outcomes (e.g., general health, periodontal health, diet changes, oral health literacy, dental anxiety, quality of life, social demographics)	8	[19, 36, 40, 44, 45, 58, 59, 63]

Participant feedback evaluated the cultural safety, acceptability and accessibility of health programmes for Aboriginal and Torres Strait Islander Peoples. Feedback was gathered from children, parents, adult participants and Aboriginal and Torres Strait Islander health staff. Surveys and questionnaires were used [24, 30, 38], although qualitative methods such as interviews or yarning were more common [23, 27, 31, 32, 33, 36, 42, 45, 57, 61, 74, 76]. Yarning fosters trust and provides a culturally safe environment for participants to share their experiences [77]. Thematic analysis was then applied to interpret the data. Additionally, the involvement of Aboriginal research staff in feedback collection further enhanced cultural safety and enriched the quality of the data gathered.

#### 4.3.2 Process Evaluation, Health Economic Evaluations

The second most commonly measured outcomes were narrative recounts of program development and implementation (7) [35, 39, 48, 49, 61, 64, 75], process evaluations (7) [25, 28, 35, 50, 59, 72, 73], and health economic evaluations (5) [29, 55, 58, 64, 69]. Narrative recounts provided insights into community co‐design and partnership methods, highlighting challenges encountered and lessons learned during program implementation. Notable examples include Baby Teeth Talk [39] and the development of a sustainable dental program for rural and remote NSW communities [28], emphasising the intense effort and time required to build strong relationships with local communities and organisations to understand community needs and answer them in a culturally safe way. Process evaluations recorded treatments provided within programs to assess if the program was carried out as planned. Economic evaluations assessed the cost‐effectiveness of interventions such as water fluoridation in remote Indigenous communities in the Northern Territory [64, 69] and remote dental services in Queensland [55, 58] and New South Wales [29].

#### 4.3.3 Other Outcomes

The least common outcomes were general health (3) [51, 52, 66], periodontal health (2) [26, 65], diet changes (2) [51, 52], oral hygiene or oral health literacy (2) [51, 52], dental anxiety (1) [70], oral health‐related quality of life (1) [70], and social demographics (1) [47]. General health links include cardiac health [66] and nutrition [51, 52].

### 4.4 Lowitja Criteria

Assessment of included studies compared to Lowitja Criteria is available in Table 2 and Table S8. Shared Responsibility, Partnerships and Engagement with Aboriginal and Torres Strait Islander Peoples and Communities were achieved by creating programs responding to community concerns [25, 61, 68], governance by Aboriginal and Torres Strait Islander reference and advisory groups [25, 28, 39, 40, 41, 44, 45, 48, 49, 50, 52, 56, 57, 67, 70, 71, 73, 75], and partnerships with Aboriginal Community Controlled Health Services (ACCHSs) [23, 24, 26, 28, 29, 30, 35, 57, 73, 75]. Regular contact and relationship building during program development and implementation ensured that programs were culturally safe and accepted by the community [39, 52].

**TABLE 2:** Lowitja criteria and related studies.

Lowitja criteria	No. of studies meeting criteria	Reference no. of studies
Shared Responsibility/Partnerships with Aboriginal and Torres Strait Islander Organisations and Communities	38	[16, 17, 18, 19, 20, 21, 22, 23, 24, 25, 26, 28, 29, 30, 31, 32, 33, 34, 36, 37, 38, 40, 41, 42, 43, 45, 46, 49, 50, 51, 54, 60, 61, 63, 64, 66, 67, 68]
Engagement with Aboriginal and Torres Strait Islander People and Communities	38	[16, 17, 18, 19, 21, 22, 23, 24, 25, 26, 28, 29, 30, 31, 32, 33, 34, 36, 37, 38, 40, 41, 42, 43, 45, 46, 48, 49, 50, 54, 60, 61, 63, 64, 66, 67, 68, 69]
Capacity Building of Aboriginal and Torres Strait Islander Communities	31	[16, 17, 18, 19, 21, 22, 23, 24, 25, 26, 28, 29, 30, 31, 32, 36, 38, 41, 42, 43, 48, 50, 54, 58, 60, 63, 64, 65, 66, 67, 68]
Equity	50	[16, 17, 18, 19, 20, 21, 22, 23, 24, 25, 26, 27, 28, 29, 30, 31, 32, 33, 34, 35, 36, 37, 38, 40, 41, 42, 43, 44, 45, 46, 48, 49, 50, 51, 52, 53, 54, 55, 57, 58, 59, 61, 62, 63, 64, 65, 66, 67, 68, 69]
Accountability	41	[16, 17, 18, 19, 20, 21, 22, 23, 24, 25, 26, 27, 28, 29, 30, 31, 32, 35, 36, 38, 40, 41, 42, 43, 46, 48, 49, 50, 52, 53, 54, 55, 57, 62, 63, 64, 65, 66, 67, 68, 69]
Evidence Based	53	[16, 17, 18, 19, 20, 21, 22, 23, 24, 25, 26, 27, 28, 29, 30, 31, 32, 33, 34, 35, 36, 37, 38, 39, 40, 41, 42, 43, 44, 45, 46, 47, 48, 49, 50, 52, 53, 54, 55, 56, 57, 58, 59, 60, 61, 62, 63, 64, 65, 66, 67, 68, 69]
Holistic Concept of Health	31	[16, 17, 19, 20, 21, 23, 24, 25, 26, 29, 30, 31, 32, 33, 34, 35, 36, 37, 38, 42, 45, 48, 50, 54, 61, 62, 63, 66, 67, 68, 69]
Cultural Competence	34	[16, 17, 18, 19, 20, 21, 22, 23, 24, 25, 26, 28, 29, 30, 31, 32, 33, 34, 36, 37, 38, 40, 41, 42, 45, 50, 54, 58, 63, 64, 66, 67, 68, 69]
Data Governance and Intellectual Property	12	[16, 17, 19, 21, 24, 25, 26, 31, 32, 45, 50, 67]
Capitalising on Indigenous Strengths	30	[16, 17, 18, 19, 21, 23, 24, 25, 26, 29, 30, 31, 32, 33, 34, 36, 37, 38, 41, 42, 45, 50, 54, 60, 63, 64, 66, 67, 68, 69]

Studies built the capacity of Aboriginal and Torres Strait Islander Communities by employing Aboriginal and Torres Strait Islander peoples as project staff [25, 26, 28, 29, 43, 48, 49, 50, 52, 55, 61, 65, 70, 71, 73, 74, 75, 76], often involved in participant communication. Some projects upskilled local community members including teachers and Aboriginal Health Workers (AHWs) [28, 32, 35, 37, 38, 50, 67] or opened dental services in Aboriginal Medical Services (AMSs) [24, 26, 29, 30, 47, 48, 57, 58].

Studies met Accountability and Evidence‐Based criteria using evidence‐based interventions, evaluation plans and data collection methods. Cultural safety was achieved by including Aboriginal and Torres Strait Islander peoples and communities as project staff in program development and implementation [23, 24, 25, 26, 27, 28, 29, 30, 31, 32, 33, 35, 36, 37, 38, 39, 40, 41, 43, 44, 45, 47, 48, 49, 52, 57, 61, 65, 70, 71, 73, 74, 75, 76] and providing cultural safety training to non‐Aboriginal and Torres Strait Islander research staff [30]. Capitalising on Indigenous strengths was done by co‐designing and including Aboriginal and Torres Strait Islander people's knowledge, utilising strong community networks [31, 32, 39, 43, 52, 61, 74], and yarning methodologies for qualitative research [25, 31, 32, 33, 45, 57, 74, 76]. Data governance, intellectual property and a holistic concept of health were the criteria that needed improvement. Only four studies had dissemination plans, such as teaching AHW staff or sharing findings with the participating community [32, 33, 39, 55].

## 5 Discussion

The Lowitja Criteria allowed assessment of the included studies and their evaluations. The discussion outlines what went well and what could be improved and embeds our findings within past research and the Australian government and mainstream dental health systems.

### 5.1 What Went Well?

Co‐design methodologies met the Lowitja Criteria of ‘Partnerships’, ‘Shared Responsibility’ and ‘Engagement with Aboriginal and Torres Strait Islander Communities’ and ‘Organisations’. Co‐designed projects should arise within communities, although externally planned projects are successful when meeting community concerns [78]. Aboriginal and Torres Strait Islander leadership should be in advisory and reference groups [78]. Partnerships reduce prejudice and bias among program evaluation members [12] and alleviate some impacts of colonisation, as Western approaches for policymaking are usually top‐down lacking community consultation [78]. An exemplar of co‐design would be Baby Teeth Talk, which benefited from strong participant retention [39]. In this program mothers were given dental care during pregnancy, and dental health counselling for their newborn children [39]. Even where disease improvement is modest, partnerships and co‐design can have profound effects ultimately improving access to care [79]. Community partnerships help communities meet their priorities, and research should not promote changes from a top‐down approach [78].

In practice, there is a lack of ‘Shared Responsibility’ between government dental services and Aboriginal Communities when partnerships are not formed with the Community, families and individuals and not just the Aboriginal Health Service. This leads to the Community being uninformed of the presence of the dental team, inadequate staffing for the dental visit, and a lack of Community capacity‐building, creating barriers in the use of dental services (per correspondence in February 2025 from J Hedges [Director, Indigenous Oral Health Unit]). Similar scenarios have been documented in the Australian health system [80]. Conversely, strong partnerships between Indigenous communities and health services strengthen traditional cultural ways of life and healing practices as seen in Canada [81].

Another feature of co‐design with Aboriginal and Torres Strait Islander peoples is that evaluators and program managers must place equal or greater value on First Nations' ways of working and perspectives [82]. One exemplar for capitalising on Indigenous strengths was AKction [45]. The research team included ‘*Kidney Warriors*’; Aboriginal people with end‐stage renal disease, who are the key participants and beneficiaries of the research project [45]. Researchers also utilised Aboriginal epistemologies including Ganma, Daddirri and clinical Yarning [45].

The inclusion of Aboriginal and Torres Strait Islander feedback is crucial for improving program effectiveness and ensuring cultural safety; however, Australian health systems lack robust mechanisms to integrate participant insights into program reorganisation [83]. The Australian Dental Association also lacks policies or guidelines on partnering with Aboriginal and Torres Strait Islander consumers [84]. The cultural safety of programs is unknown without this feedback [85]. Mainstream dental services are often not culturally safe [86]; thus, Aboriginal and Torres Strait Islander dental program evaluations must identify factors that enhance cultural safety for broader implementation across diverse settings.

### 5.2 What Could Be Improved?

#### 5.2.1 Study Designs

More high‐quality evaluations are needed to meet the cultural and academic standards of Indigenous Peoples [12], as reflected in the Lowitja Criteria of ‘Evidence‐Based’ and ‘Accountability’ [10]. Mixed methods are preferred to measure the biomedical outcomes of health programmes and programme utility and impact on the community [8]. Some studies reported not using an RCT study design due to ethical concerns [60]. However, multiple studies have been conducted using this design ethically [40, 41, 43, 44, 51, 52, 63, 65, 66]. Findings from these studies are more rigorous [87], and are taken more seriously by decision‐makers [88].

When RCTs are not feasible, especially for community work, alternative high‐quality study designs are crucial to ensure robust and reliable findings [89]. In these cases, it is recommended by international Indigenous epidemiology researchers to choose the study design best aligned to community experiences [90]. One study with design limitations comes from the Northern Territory, where researchers attributed differences in dental caries between remote communities solely to water fluoridation [69]. This approach failed to account for other critical social determinants of health, such as socioeconomic status (SES), proximity to major towns with better access to dental and healthcare services and the availability of health services [91]. A case–control study design would have been better to compare the effects of community water fluoridation between similar communities.

#### 5.2.2 Holistic Concept of Health

Good dental health includes optimal chewing and speech, absence or prevention of pain, confidence in aesthetics, not having anxiety about dental health and satisfaction with dental treatment [92]. Any programme that improves these is successful from a participant perspective even if there is only a modest reduction in caries. Improvements in dental‐related quality of life should be measured within the Aboriginal and Torres Strait Islander Holistic Concept of Health framework which includes family, community and societal wellbeing [93]. Considering dental health within broader well‐being is also promoted in the dentistry community [94]. An exemplar of measurement of holistic health was done in the Kimberley region where researchers measured decreases in caries [71], decreases in dental anxiety and improvements in oral‐health‐related quality of life [70].

Australian government dental systems do not assess outcome or impact measures but only report the volume of treatments provided to measure their success [18]. The Victorian government dental system is pivoting to capturing outcome measures to assess the effectiveness of its dental services [95]. This trend should continue in other states. For Aboriginal and Torres Strait Islander dental programmes, impact and outcome measures must be culturally relevant [85] and reflect local Indigenous understandings of wellbeing [12].

#### 5.2.3 Long‐Term Sustainable Improvements

The most important Lowitja Criteria to be improved upon is ‘Data Governance’ and ‘Intellectual Property’, which was unclear in 41 of 55 included studies (75%). There are notable examples of effective interventions that government dental systems have not taken up, for example, Baby Teeth Talk in SA [44], Motivational Interviewing and Atratumatic Restorative Technique and Hall Technique (MI and ART‐HT) in Kimberleys [70]. The Lowitja Institute recognises that ‘[there is an] independent manner in which the researchers and decision‐makers operate’ [10]. Government support can suddenly be taken away as experienced by the Pika Wiya Dental Service [48]. Researchers partnered with the local community and state government dental services to open a dental clinic within a regional town's Aboriginal Medical Service (AMS). This clinic was more culturally safe than local government clinics [49]. At present, this has now closed; the school dentist is closed, and the government dentist in town is stretched thin across prison, general anaesthetic and neighbouring town clinics (per email correspondence in January 2025 from Dr. N. Hiku [Dental Officer, Mid North‐Yorke, SA Dental]). Indigenous health programs are often at the mercy of government support; in New Zealand the Te Aka Whai Ora (the Māori Health Authority) invested in local capacity building and Maori health research, and it's disestablishment in 2024 will revoke these gains in health [96].

For long‐term sustainable improvements to dental health for Aboriginal and Torres Strait Islander Peoples, adequate resources must be allocated to project and evaluation staff [8]. Resources can include human resources, cultural expertise, time, monetary compensation for community participation and engagement, and relationship building [12]. This support ensures that Aboriginal Communities and organisations have the capacity to implement sustainable and culturally safe dental programmes.

### 5.3 Strengths and Limitations

The key strength of this research was the embedding of Aboriginal and Torres Strait Islander values into the research findings and discussion. The Lowitja Framework allowed for reliable analyses of studies by breaking down important factors for Aboriginal and Torres Strait Islander health program evaluations into simplified criteria. There were many included studies across different states, which permit these findings to be generalised to the many Aboriginal and Torres Strait Islander communities. The Lowitja Framework was designed for Aboriginal and Torres Strait Islander people in Australia and therefore cannot be directly utilised in international environments. However, it does emphasise the importance of community and cultural values to judge the success of an Indigenous health programme. This review demonstrates how culturally informed definitions of health can be utilised in dental health research. International researchers and health managers can develop local frameworks with Indigenous input to shift focus away from evaluations of solely clinical indicators and ensure that culturally relevant factors of health programmes are identified and strengthened.

A limitation of this systematic review is that the Lowitja Framework was based on evaluations via peer reviewed literature and tenders [10] and this review only included peer reviewed literature. Different sampling was required because government dental systems do not often collect or report outcome measures of their programs [18]. This could affect the findings because published research may measure and report clinical effectiveness more often than community or societal outcomes [97]. For example, in the Smiles not Tears published research [38], there was no mention of an Aboriginal advisory group; however, in the unpublished thesis, the author does detail this [98]. This risks underestimation of program evaluations meeting the Lowitja Criteria.

Another limitation is that the Lowitja Framework has explicit indicators for meeting criteria [10]. If this data was unavailable, more subjective indicators were used, linking the Lowitja framework's descriptions of their criteria with Supporting Information S1 in studies. This risks over‐estimation of programme evaluations meeting the criteria and decreases reliability.

## 6 Conclusion

Aboriginal and Torres Strait Islander dental evaluations followed most Lowitja Framework criteria, especially shared responsibility, partnerships and engagement with Aboriginal and Torres Strait Islander peoples and communities. The Lowitja Framework highlights the success that strong partnerships achieve, turning away from biomedical definitions of health programme success. Strong partnerships and community feedback improved cultural safety. Appropriate study designs must be used, and culturally relevant and holistic outcome measures chosen to evaluate dental programmes. The Lowitja Framework and more culturally relevant outcomes could be present in upstream and downstream dental systems. Health departments and funding managers could introduce indicators for cultural safety, community engagement and Aboriginal governance. On‐the‐ground dental clinic managers, clinicians and assistants can collect feedback from community members and advocate upwards for policy change. The findings of this review can be utilised to improve the effectiveness of Aboriginal and Torres Strait Islander dental programme evaluations.

## Author Contributions

A.P., J.H., L.J. and S.N. all contributed to the planning, conduct and reporting of the work described in the manuscript. A.P. conceived the presented idea. A.P., L.J. and S.N. devised the methodology of this paper. A.P., S.N. and L.J. performed the search of the databases for eligible studies and full‐text assessment, methodological assessment, data extraction and data entry. A.P., J.H., L.J. and S.N. contributed to the interpretation and analysis of the results. S.N., A.P. and L.J. wrote this paper. All authors contributed to the writing up, review, editing and finalising of the manuscript.

## Ethics Statement

The Aboriginal Health Council of South Australia Research Ethics Committee does not require ethical approval for systematic reviews [99].

## Conflicts of Interest

The authors declare no conflicts of interest.

## Supporting information


**Data S1:** cdoe70023‐sup‐0001‐supinfo.docx.

## Data Availability

Data supporting the findings of this study are available within the article and Supporting Information S1. Further data are available from the corresponding author, AP, upon reasonable request.
