# Supplementary material for: Aboriginal and Torres Strait Islander Dental Program Evaluations: A Mixed‐Methods Systematic Review
Source: Community Dent Oral Epidemiol. 2025 Aug 27;53(6):713–23. doi: 10.1111/cdoe.70023 (PMC12627262; doi:10.1111/cdoe.70023)
Supplement: Supplementary file 1 — Data S1: cdoe70023‐sup‐0001‐supinfo.docx. [file CDOE-53-713-s001.docx]

Appendix-Supplementary Materials

Contents

[Supplementary tables 1](#_Toc332145315)

[Table S1: PRISMA 2020 Abstract1 2](#_Toc501779033)

[Table S2: Prisma 2020 Checklist1 3](#_Toc1994618516)

[Table S3: Inclusion and Exclusion criteria based on the SPIDER framework2 8](#_Toc536932234)

[Table S4: Search Strategy 10](#_Toc630592811)

[Table S5. Excluded studies 13](#_Toc2059488905)

[Table S6: QuADS Critical Appraisal 15](#_Toc1405637530)

[Table S7: Characteristics of Included Studies 20](#_Toc198246671)

[Table S8: Included Studies Assessed with Lowitja Criteria 28](#_Toc1749367080)

[Supplementary Figures 48](#_Toc927938645)

[Figure S1: Histogram of Average QuADS Score and Number of Studies 48](#_Toc128552420)

# Supplementary tables

## Table S1: PRISMA 2020 Abstract^1^

| **Section and Topic** | **Item #** | **Checklist item** | **Reported (Yes/No)** |
| --- | --- | --- | --- |
| **TITLE** | | |  |
| Title | 1 | Identify the report as a systematic review. | Yes |
| **BACKGROUND** | | |  |
| Objectives | 2 | Provide an explicit statement of the main objective(s) or question(s) the review addresses. | Yes |
| **METHODS** | | |  |
| Eligibility criteria | 3 | Specify the inclusion and exclusion criteria for the review. | Yes |
| Information sources | 4 | Specify the information sources (e.g. databases, registers) used to identify studies and the date when each was last searched. | Yes |
| Risk of bias | 5 | Specify the methods used to assess risk of bias in the included studies. | Yes |
| Synthesis of results | 6 | Specify the methods used to present and synthesise results. | Yes |
| **RESULTS** | | |  |
| Included studies | 7 | Give the total number of included studies and participants and summarise relevant characteristics of studies. | Yes |
| Synthesis of results | 8 | Present results for main outcomes, preferably indicating the number of included studies and participants for each. If meta-analysis was done, report the summary estimate and confidence/credible interval. If comparing groups, indicate the direction of the effect (i.e. which group is favoured). | Yes |
| **DISCUSSION** | | |  |
| Limitations of evidence | 9 | Provide a brief summary of the limitations of the evidence included in the review (e.g. study risk of bias, inconsistency and imprecision). | Yes |
| Interpretation | 10 | Provide a general interpretation of the results and important implications. | Yes |
| **OTHER** | | |  |
| Funding | 11 | Specify the primary source of funding for the review. | N/A - no funding |
| Registration | 12 | Provide the register name and registration number. | Yes |

## Table S2: Prisma 2020 Checklist^1^

| **Section and Topic** | **Item #** | **Checklist item** | **Location where item is reported** |
| --- | --- | --- | --- |
| **TITLE** | | |  |
| Title | 1 | Identify the report as a systematic review. | Title |
| **ABSTRACT** | | |  |
| Abstract | 2 | See the PRISMA 2020 for Abstracts checklist. | Table S1 |
| **INTRODUCTION** | | |  |
| Rationale | 3 | Describe the rationale for the review in the context of existing knowledge. | 1. Introduction |
| Objectives | 4 | Provide an explicit statement of the objective(s) or question(s) the review addresses. | 1. Introduction |
| **METHODS** | | |  |
| Eligibility criteria | 5 | Specify the inclusion and exclusion criteria for the review and how studies were grouped for the syntheses. | 2.1. Eligibility Criteria |
| Information sources | 6 | Specify all databases, registers, websites, organisations, reference lists and other sources searched or consulted to identify studies. Specify the date when each source was last searched or consulted. | 2.3. Search Strategy and Database Selection |
| Search strategy | 7 | Present the full search strategies for all databases, registers and websites, including any filters and limits used. | Table S4 |
| Selection process | 8 | Specify the methods used to decide whether a study met the inclusion criteria of the review, including how many reviewers screened each record and each report retrieved, whether they worked independently, and if applicable, details of automation tools used in the process. | 2.4. Study Selection |
| Data collection process | 9 | Specify the methods used to collect data from reports, including how many reviewers collected data from each report, whether they worked independently, any processes for obtaining or confirming data from study investigators, and if applicable, details of automation tools used in the process. | 2.6. Data Extraction and 2.7 Data Transformation, Synthesis, Integration and Analysis |
| Data items | 10a | List and define all outcomes for which data were sought. Specify whether all results that were compatible with each outcome domain in each study were sought (e.g. for all measures, time points, analyses), and if not, the methods used to decide which results to collect. | S7 & S8 |
|  | 10b | List and define all other variables for which data were sought (e.g. participant and intervention characteristics, funding sources). Describe any assumptions made about any missing or unclear information. | S7 |
| Study risk of bias assessment | 11 | Specify the methods used to assess risk of bias in the included studies, including details of the tool(s) used, how many reviewers assessed each study and whether they worked independently, and if applicable, details of automation tools used in the process. | 2.5 Assessment of Methodological Quality 4.1 Methodological Quality and |
| Effect measures | 12 | Specify for each outcome the effect measure(s) (e.g. risk ratio, mean difference) used in the synthesis or presentation of results. | N/A |
| Synthesis methods | 13a | Describe the processes used to decide which studies were eligible for each synthesis (e.g. tabulating the study intervention characteristics and comparing against the planned groups for each synthesis (item #5)). | 2.6. Data Extraction and 2.7 Data Transformation, Synthesis, Integration and Analysis |
|  | 13b | Describe any methods required to prepare the data for presentation or synthesis, such as handling of missing summary statistics, or data conversions. | 2.7 Data Transformation, Synthesis, Integration and Analysis |
|  | 13c | Describe any methods used to tabulate or visually display results of individual studies and syntheses. | 2.6. Data Extraction and 2.7 Data Transformation, Synthesis, Integration and Analysis |
|  | 13d | Describe any methods used to synthesize results and provide a rationale for the choice(s). If meta-analysis was performed, describe the model(s), method(s) to identify the presence and extent of statistical heterogeneity, and software package(s) used. | 2.7 Data Transformation, Synthesis, Integration and Analysis |
|  | 13e | Describe any methods used to explore possible causes of heterogeneity among study results (e.g. subgroup analysis, meta-regression). | N/A |
|  | 13f | Describe any sensitivity analyses conducted to assess robustness of the synthesized results. | N/A |
| Reporting bias assessment | 14 | Describe any methods used to assess risk of bias due to missing results in a synthesis (arising from reporting biases). | N/A |
| Certainty assessment | 15 | Describe any methods used to assess certainty (or confidence) in the body of evidence for an outcome. | n/A |
| **RESULTS** | | |  |
| Study selection | 16a | Describe the results of the search and selection process, from the number of records identified in the search to the number of studies included in the review, ideally using a flow diagram. | 3.1 Study Inclusion |
|  | 16b | Cite studies that might appear to meet the inclusion criteria, but which were excluded, and explain why they were excluded. | Table S5 |
| Study characteristics | 17 | Cite each included study and present its characteristics. | Table S7 |
| Risk of bias in studies | 18 | Present assessments of risk of bias for each included study. | Table S6 |
| Results of individual studies | 19 | For all outcomes, present, for each study: (a) summary statistics for each group (where appropriate) and (b) an effect estimate and its precision (e.g. confidence/credible interval), ideally using structured tables or plots. | N/A |
| Results of syntheses | 20a | For each synthesis, briefly summarise the characteristics and risk of bias among contributing studies. | N/A |
|  | 20b | Present results of all statistical syntheses conducted. If meta-analysis was done, present for each the summary estimate and its precision (e.g. confidence/credible interval) and measures of statistical heterogeneity. If comparing groups, describe the direction of the effect. | N/A |
|  | 20c | Present results of all investigations of possible causes of heterogeneity among study results. | N/A |
|  | 20d | Present results of all sensitivity analyses conducted to assess the robustness of the synthesized results. | N/A |
| Reporting biases | 21 | Present assessments of risk of bias due to missing results (arising from reporting biases) for each synthesis assessed. | N/A |
| Certainty of evidence | 22 | Present assessments of certainty (or confidence) in the body of evidence for each outcome assessed. | N/A |
| **DISCUSSION** | | |  |
| Discussion | 23a | Provide a general interpretation of the results in the context of other evidence. | 5. Discussion |
|  | 23b | Discuss any limitations of the evidence included in the review. | 5.3 Strengths and Limitations |
|  | 23c | Discuss any limitations of the review processes used. | 5.3 Strengths and Limitations |
|  | 23d | Discuss implications of the results for practice, policy, and future research. | 5. Discussion |
| **OTHER INFORMATION** | | |  |
| Registration and protocol | 24a | Provide registration information for the review, including register name and registration number, or state that the review was not registered. | 2. Method |
|  | 24b | Indicate where the review protocol can be accessed, or state that a protocol was not prepared. | 2. Method |
|  | 24c | Describe and explain any amendments to information provided at registration or in the protocol. | N/A - no changes |
| Support | 25 | Describe sources of financial or non-financial support for the review, and the role of the funders or sponsors in the review. | Funding Statement |
| Competing interests | 26 | Declare any competing interests of review authors. | Conflicts of Interest |
| Availability of data, code and other materials | 27 | Report which of the following are publicly available and where they can be found: template data collection forms; data extracted from included studies; data used for all analyses; analytic code; any other materials used in the review. | Data Availability |

## Table S3: Inclusion and Exclusion criteria based on the SPIDER framework^2^

| **Category** | **Inclusion** | **Exclusion** |
| --- | --- | --- |
| Samples (S) | Aboriginal and Torres Strait Islander participants |  |
| Phenomenon of interest (PI) | Dental health programs and their evaluations | Dental programs that were not explicitly developed with and for Aboriginal and Torres Strait Islander Peoples |
| Design (D) | - Quantitative descriptive research   - Incidence or prevalence study without comparison group   - Survey   - Grounded theory   - Ethnography research   - Case studies   - Observational studies   - Phenomenological research   - Narrative research   - Qualitative description   - Mixed methods design | - Literature reviews including systematic or scoping reviews  - Study protocols |
| Evaluation (E) | Perspectives, views, attitudes, preferences, cultural safety, effectiveness, experiences |  |
| Research type (R) | Studies published in peer-reviewed journals:  Qualitative, quantitative, or mixed methods research |  |

## Table S4: Search Strategy

Aboriginal and Torres Strait Islander Peoples are referenced in literature by the term “Aboriginal” and the state or country of residence. The term “Torres Strait Islander” has been used alone. Other terms used are “indigenous”, “first nation”, or “native people”. Specifying Australia and Australian states excludes studies of Indigenous Peoples from other countries. “Dental Care” can be identified by reference to the personnel involved in the study, including dental assistant, dental hygienist, dental or oral health therapist, or dentist. There may also be references to dentistry, dental or oral health.

| **Aboriginal and Torres Strait Islander Peoples** | **Dental care** |
| --- | --- |
| PubMed | |
| "Australian Aboriginal and Torres Strait Islander Peoples"[mh]  OR "Native Hawaiian or Other Pacific Islander"[mh] OR  ((Aborigin*[tiab] OR indigenous[tiab] OR First Nations[tiab]  OR Native people*[tiab]) AND (Australia*[tiab] OR Northern  Territory[tiab] OR Tasmania*[tiab] OR New South Wales[tiab]  OR Victoria*[tiab] OR Queensland*[tiab]) OR Torres Strait Islander*[tiab]) | Dentist*[tiab] OR  Dental[tiab] OR  Oral health*[tiab]    OR    "Oral Health"[mh] OR  "Dentistry"[mh] |
| Scopus | |
| TITLE-ABS-KEY((Australia* OR "Northern Territory" OR Tasmania* OR "New South Wales" OR Victoria* OR Queensland*) AND (Aborigin* OR indigenous OR "First Nations" OR "Native people*")) OR "Torres Strait Islander*" | TITLE-ABS-KEY(Dentist* or dental or "oral health") |
| Embase | |
| Exp indigenous australian or (indigenous or aborigin* or first nation*) and (australia* or “northern territory” or tasmania or “new south wales” or victoria* or queensland*) or “torres strait islander*”.ti,ab | (dentist* or dental or oral health*).ti,ab |
| Dentistry & Oral Sciences Source (DOSS) | |
| DE "INDIGENOUS Australians" OR DE "ABORIGINAL Australians" OR DE "TORRES Strait Islanders" OR TI (((Australia* OR "Northern Territory" OR Tasmania* OR "New South Wales" OR Victoria* OR Queensland*) AND (Aborigin* OR indigenous OR "First Nations" OR "Native people*")) OR "Torres Strait Islander*") OR AB (((Australia* OR "Northern Territory" OR Tasmania* OR "New South Wales" OR Victoria* OR Queensland*) AND (Aborigin* OR indigenous OR "First Nations" OR "Native people*")) OR "Torres Strait Islander*") | DE “DENTAL care” OR TI (dentist* OR “dental” or “oral health”) OR AB (dentist* OR “dental” or “oral health”) |
| Public Health Database | |
| ABSTRACT,TITLE((((indigenous or aborigin* or (first nation*)) or (native people*) and australia* or (northern territory) or tasmania* or (new south wales) or victoria* or queensland*) or (torres strait islander*)) and (dental* or (oral health) or dentist*)) |  |

## Table S5. Excluded studies

| **Study Reference** | **Reason for exclusion** |
| --- | --- |
| Arrow P. Service Use and Perceived Need among an Aboriginal Population in Western Australia. Journal of Health Care for the Poor and Underserved. 2016;27(1):90-100. | Wrong study design - insufficient focus on specific dental program |
| Arrow P, McPhee R, Atkinson D, et al. Minimally Invasive Dentistry Based on Atraumatic Restorative Treatment to Manage Early Childhood Caries in Rural and Remote Aboriginal Communities: Protocol for a Randomized Controlled Trial. *JMIR Research Protocols.* 2018;7(7):n/a. | Wrong study design - study protocol |
| Bailie RS, Stevens M, Armfield JM, Ehsani JP, Beneforti M, Spencer J. Association of natural fluoride in community water supplies with dental health of children in remote Indigenous communities - Implications for policy. *Australian and New Zealand Journal of Public Health.* 2009;33(3):205-211. | Wrong intervention - no program implemented |
| Blinkhorn F, Brown N, Freeman R, Humphris G, Martin A, Blinkhorn A. A phase II clinical trial of a dental health education program delivered by aboriginal health workers to prevent early childhood caries. *BMC Public Health.* 2012;12:681. | Secondary data (e.g. study protocol) - study protocol |
| Dimitropoulos Y, Gunasekera H, Blinkhorn A, et al. A collaboration with local Aboriginal communities in rural New South Wales, Australia to determine the oral health needs of their children and develop a community-owned oral health promotion program. *Rural and Remote Health.* 2018;18(2):4453. | Wrong intervention - no program implemented |
| Dimitropoulos Y, Holden A, Gwynne K, Irving M, Binge N, Blinkhorn A. An assessment of strategies to control dental caries in Aboriginal children living in rural and remote communities in New South Wales, Australia. *BMC Oral Health.* 2018;18(1):N.PAG-N.PAG. | Secondary data (e.g. study protocol) - no program implemented |
| Feain D. Oral care gets teeth; Campaign targeting health of Aboriginal youngsters. *The Northern Star.* 2011:1. | Secondary data (e.g. study protocol) - news article |
| Gao Y, Ju X, Jamieson L. Associations between dental care approachability and dental attendance among women pregnant with an Indigenous child: a cross-sectional study. *BMC Oral Health.* 2021;21(1):1-10. | Wrong study design - insufficient focus on specific dental program |
| Hedges J, Gustavo Hermes S, Cadet-James Y, et al. A Silver Fluoride Intervention to Improve Oral Health Trajectories of Young Indigenous Australians: Protocol for a Cluster Randomized Controlled Trial. *JMIR Research Protocols.* 2023;12:e48558. | Secondary data (e.g. study protocol) - study protocol |
| Irving M, Kumar N, Gwynne K, Talbot F, Blinkhorn AS. Improving oral-health-related quality-of-life for rural Aboriginal communities in Australia utilising a novel mobile denture service. *Rural and Remote Health.* 2019;19(3):5063. | Secondary data (e.g. study protocol) – research letter |
| Jamieson L, Skilton M, Maple-Brown L, et al. Periodontal disease and chronic kidney disease among Aboriginal adults; An RCT. *BMC Nephrology.* 2015;16(1):181. | Secondary data (e.g. study protocol) – study protocol |
| Lalloo R, Kroon J, Tut O, et al. Effectiveness, cost-effectiveness and cost-benefit of a single annual professional intervention for the prevention of childhood dental caries in a remote rural Indigenous community. *BMC Oral Health.* 2015;15(1):1-8. | Secondary data (e.g. study protocol) – study protocol |
| Lawrence HP, Binguis D, Douglas J, et al. A 2-year community-randomized controlled trial of fluoride varnish to prevent early childhood caries in Aboriginal children. *Community Dentistry and Oral Epidemiology.* 2008;36(6):503. | Wrong setting – Canada |
| McAuliffe A, Bourke C, Jamieson LM. Addressing the oral health needs of Indigenous Australians through water fluoridation. Medical Journal of Australia. 2020;213(6):286-286.e281. | Secondary data (e.g. study protocol) – research letter |
| Merrick J, Chong A, Parker E, et al. Reducing disease burden and health inequalities arising from chronic disease among Indigenous children: an early childhood caries intervention. *BMC Public Health.* 2012;12:323. | Secondary data (e.g. study protocol) – study protocol |
| Parker EJ, Misan G, Chong A, et al. An oral health literacy intervention for Indigenous adults in a rural setting in Australia. *BMC Public Health.* 2012;12:461. | Secondary data (e.g. study protocol) – study protocol |
| Patel J, Durey A, Naoum S, Kruger E, Slack-Smith L. Oral health education and prevention strategies among remote Aboriginal communities: a qualitative study. *Australian Dental Journal.* 2022;67(1):83-93. | Wrong study design - insufficient focus on specific dental program |
| Patel J, Nattabi B, Long R, et al. The 5C model: A proposed continuous quality improvement framework for volunteer dental services in remote Australian Aboriginal communities. *Community Dentistry and Oral Epidemiology.* 2023;51(6):1150-1158. | Wrong study design - insufficient focus on specific dental program |
| Poirier B, Tang S, Haag DG, Sethi S, Hedges J, Jamieson L. Oral health promotion and programming provided by Aboriginal Community Controlled Health Organisations in South Australia. *Health promotion journal of Australia : official journal of Australian Association of Health Promotion Professionals.* 2022;33(Supplement 1):255-261. | Wrong study design - insufficient focus on specific dental program |
| Poirier BF, Hedges J, Smithers LG, Moskos M, Jamieson LM. Child-, Family-, and Community-Level Facilitators for Promoting Oral Health Practices among Indigenous Children. *International Journal of Environmental Research and Public Health.* 2022;19(3):1150. | Wrong study design - insufficient focus on specific dental program |
| Skilton MR, Maple-Brown LJ, Kapellas K, et al. The effect of a periodontal intervention on cardiovascular risk markers in Indigenous Australians with periodontal disease: the PerioCardio study. *BMC Public Health.* 2011;11:729. | Secondary data (e.g. study protocol) – study protocol |
| Tynan A, Walker D, Tucker T, Fisher B, Fisher T. Managing oral health care and prevention: The experience of Aboriginal and Torres Strait Islanders living in a rural community in Queensland, Australia. *The Australian journal of rural health.* 2022;30(2):228-237. | Wrong study design - insufficient focus on specific dental program |
| Ummer-Christian R, Widdicombe D, Raichur A, Couch D. Aboriginal Health Practitioners obtaining, possessing and administering fluoride varnish: self-determination driven regulation amendment for integrated oral health care for Aboriginal children. *Australian Journal of Primary Health.* 2024;30(1):PY23201. | Wrong study design – research letter |
| Jamieson LM, Smithers LG, Hedges J, et al. Follow-up of an Intervention to Reduce Dental Caries in Indigenous Australian Children: A Secondary Analysis of a Randomized Clinical Trial. *JAMA Network Open.* 2019;2(3):e190648. | Duplicate |

## Table S6: QuADS Critical Appraisal

| Author, Year | 1. Theoretical or conceptual underpinning to the research | 2. Statement of research aim/s | 3. Clear description of research setting and target population | 4. The study design is appropriate to address the stated research aim/s | 5. Appropriate sampling to address the research aim/s | 6. Rationale for choice of data collection tool/s | 7. The format and content of data collection tool is appropriate to address the stated research aim/s | 8. Description of data collection procedure | 9. Recruitment data provided | 10. Justification for analytic method selected | 11. The method of analysis was appropriate to answer the research aim/s | 12. Evidence that the research stakeholders have been considered in research design or conduct | 13. Strengths and limitations critically discussed | Average |
| --- | --- | --- | --- | --- | --- | --- | --- | --- | --- | --- | --- | --- | --- | --- |
| Smith et al. , 2016 | 2 | 2 | 2 | 2 | 2 | 2 | 3 | 2 | 1 | 2 | 3 | 0 | 0 | 1.8 |
| Smith et al., 2018 | 2 | 3 | 3 | 2 | 3 | 3 | 3 | 3 | 1 | 3 | 2 | 3 | 3 | 2.6 |
| Smith et al., 2020 | 2 | 2 | 2 | 3 | 1 | 1 | 2 | 1 | 2 | 0 | 1 | 0 | 0 | 1.3 |
| Patel et al., 2015 | 3 | 3 | 3 | 3 | 2 | 1 | 3 | 3 | 2 | 2 | 3 | 1 | 3 | 2.5 |
| Patel et al., 2023 | 3 | 3 | 3 | 3 | 1 | 3 | 3 | 2 | 2 | 3 | 3 | 1 | 3 | 2.5 |
| Patel et al., 2023 | 2 | 3 | 3 | 3 | 1 | 3 | 3 | 0 | 0 | 3 | 3 | 3 | 1 | 2.2 |
| Patel et al., 2021 | 3 | 3 | 3 | 3 | 3 | 3 | 3 | 3 | 3 | 2 | 3 | 3 | 3 | 2.9 |
| Jamieson et al., 2018 | 3 | 3 | 3 | 3 | 3 | 2 | 3 | 2 | 3 | 3 | 3 | 3 | 3 | 2.8 |
| Jamieson et al., 2019 | 2 | 2 | 3 | 3 | 2 | 3 | 3 | 3 | 3 | 3 | 3 | 3 | 2 | 2.7 |
| Ju et al., 2023 | 3 | 3 | 3 | 3 | 3 | 3 | 3 | 3 | 3 | 3 | 3 | 3 | 3 | 3 |
| Hammersley et al., 2022 | 3 | 3 | 3 | 3 | 3 | 3 | 3 | 3 | 3 | 3 | 3 | 3 | 3 | 3 |
| Smithers et al., 2021 | 2 | 3 | 3 | 3 | 3 | 3 | 3 | 3 | 3 | 3 | 3 | 3 | 2 | 2.8 |
| Smithers et al., 2017 | 3 | 3 | 3 | 3 | 3 | 3 | 3 | 3 | 3 | 3 | 3 | 3 | 3 | 3 |
| Kularatna et al., 2020 | 2 | 1 | 3 | 3 | 3 | 3 | 3 | 3 | 3 | 3 | 3 | 3 | 2 | 2.7 |
| Lalloo et al., 2021 | 2 | 3 | 3 | 3 | 2 | 3 | 3 | 3 | 2 | 3 | 3 | 3 | 1 | 2.6 |
| Tadakamadla et al., 2020 | 3 | 3 | 3 | 3 | 2 | 3 | 3 | 3 | 2 | 3 | 3 | 1 | 2 | 2.6 |
| Kroon et al., 2019 | 2 | 3 | 3 | 2 | 1 | 3 | 3 | 3 | 2 | 2 | 3 | 1 | 1 | 2.2 |
| Divaris et al., 2013 | 1 | 3 | 3 | 3 | 3 | 3 | 3 | 3 | 2 | 3 | 3 | 0 | 1 | 2.4 |
| Slade et al., 2011 | 3 | 2 | 3 | 3 | 3 | 3 | 3 | 3 | 3 | 3 | 3 | 3 | 3 | 2.9 |
| Spencer et al., 2010 | 3 | 1 | 3 | 3 | 1 | 3 | 3 | 3 | 1 | 3 | 3 | 3 | 3 | 2.5 |
| Zhao et al., 2023 | 3 | 3 | 3 | 2 | 2 | 2 | 3 | 1 | 1 | 3 | 3 | 0 | 2 | 2.2 |
| Chondur et al., 2024 | 3 | 2 | 3 | 2 | 2 | 2 | 2 | 1 | 1 | 3 | 2 | 0 | 2 | 1.9 |
| Ehsani & Bailie, 2007 | 2 | 3 | 3 | 3 | 0 | 3 | 3 | 3 | 0 | 3 | 3 | 3 | 0 | 2.2 |
| Arrow et al., 2020 | 3 | 3 | 3 | 3 | 3 | 3 | 3 | 3 | 3 | 3 | 3 | 3 | 3 | 3 |
| Piggott et al., 2021 | 1 | 3 | 3 | 3 | 3 | 3 | 3 | 3 | 1 | 2 | 2 | 3 | 3 | 2.5 |
| Arrow et al., 2022 | 3 | 3 | 3 | 3 | 3 | 3 | 3 | 3 | 3 | 3 | 3 | 3 | 2 | 2.9 |
| Kapellas et al., 2013 | 3 | 2 | 3 | 3 | 3 | 3 | 3 | 3 | 3 | 3 | 3 | 1 | 3 | 2.8 |
| Kapellas et al., 2014 | 1 | 3 | 3 | 3 | 2 | 3 | 3 | 3 | 2 | 3 | 3 | 1 | 1 | 2.4 |
| Parker et al., 2005 | 3 | 2 | 3 | 3 | 0 | 0 | 3 | 0 | 0 | 0 | 3 | 3 | 3 | 1.8 |
| Parker & Jamieson, 2007 | 1 | 3 | 3 | 3 | 2 | 3 | 3 | 2 | 2 | 3 | 3 | 2 | 2 | 2.5 |
| Jamieson, Parker & Richards, 2008 | 2 | 3 | 3 | 3 | 2 | 3 | 3 | 2 | 2 | 3 | 2 | 3 | 3 | 2.6 |
| Parker et al., 2012 | 3 | 3 | 3 | 3 | 1 | 3 | 3 | 3 | 3 | 3 | 3 | 3 | 3 | 2.8 |
| March et al., 2023 | 3 | 3 | 3 | 3 | 2 | 3 | 3 | 3 | 1 | 3 | 3 | 3 | 1 | 2.6 |
| Mangoyana et al., 2022 | 3 | 3 | 3 | 3 | 2 | 3 | 3 | 2 | 3 | 3 | 3 | 3 | 3 | 2.8 |
| Kelly et al., 2024 | 3 | 1 | 3 | 3 | 2 | 3 | 3 | 3 | 2 | 3 | 3 | 3 | 2 | 2.6 |
| Kong et al., 2020 | 3 | 3 | 3 | 3 | 3 | 3 | 3 | 3 | 2 | 3 | 3 | 3 | 3 | 2.9 |
| Kong et al., 2021 | 3 | 2 | 3 | 3 | 3 | 3 | 3 | 3 | 3 | 3 | 3 | 3 | 3 | 2.9 |
| Kong et al., 2021 | 3 | 3 | 3 | 3 | 2 | 3 | 3 | 3 | 1 | 3 | 3 | 3 | 2 | 2.7 |
| Kruger et al., 2010 | 2 | 3 | 3 | 3 | 3 | 3 | 3 | 2 | 2 | 3 | 3 | 1 | 1 | 2.5 |
| Roberts-Thomson et al., 2019 | 3 | 2 | 2 | 3 | 2 | 1 | 2 | 0 | 1 | 0 | 1 | 0 | 2 | 1.5 |
| Roberts-Thomson et al., 2010 | 3 | 3 | 2 | 3 | 1 | 3 | 3 | 3 | 2 | 2 | 3 | 3 | 3 | 2.6 |
| Skinner et al., 2020 | 3 | 3 | 3 | 3 | 3 | 3 | 3 | 3 | 3 | 2 | 3 | 3 | 3 | 2.9 |
| Stormon, 2022 | 3 | 3 | 3 | 3 | 2 | 3 | 3 | 3 | 2 | 1 | 3 | 1 | 1 | 2.4 |
| Johnson et al., 2014 | 3 | 1 | 3 | 3 | 2 | 3 | 3 | 3 | 2 | 3 | 3 | 2 | 3 | 2.6 |
| Dimitropoulos et al., 2020 | 3 | 3 | 3 | 2 | 1 | 1 | 2 | 3 | 2 | 2 | 3 | 3 | 3 | 2.4 |
| Dimitropoulos et al, 2017 | 3 | 3 | 3 | 2 | 2 | 3 | 2 | 3 | 1 | 3 | 2 | 3 | 3 | 2.5 |
| Dimitropoulos et al., 2020 | 3 | 3 | 3 | 3 | 2 | 2 | 2 | 2 | 2 | 2 | 3 | 3 | 3 | 2.5 |
| Dimitropoulos et al., 2019 | 3 | 2 | 3 | 3 | 2 | 2 | 3 | 3 | 1 | 3 | 3 | 3 | 3 | 2.6 |
| Lalloo et al., 2015 | 3 | 2 | 3 | 3 | 2 | 3 | 3 | 3 | 1 | 3 | 3 | 2 | 2 | 2.5 |
| Gwynne et al., 2017 | 3 | 3 | 3 | 3 | 1 | 2 | 3 | 3 | 1 | 2 | 3 | 3 | 1 | 2.4 |
| Gwynne et al., 2016 | 3 | 0 | 3 | 0 | 0 | 0 | 0 | 0 | 0 | 0 | 0 | 3 | 0 | 0.7 |
| Irving et al., 2017 | 3 | 3 | 3 | 3 | 2 | 3 | 3 | 3 | 2 | 3 | 3 | 3 | 3 | 2.8 |
| Walker et al., 2022 | 3 | 2 | 3 | 3 | 3 | 3 | 3 | 3 | 3 | 3 | 3 | 3 | 3 | 2.9 |
| Campbell et al., 2015 | 3 | 2 | 3 | 3 | 2 | 3 | 3 | 3 | 2 | 3 | 3 | 3 | 1 | 2.6 |
| Ju et al., 2017 | 3 | 3 | 3 | 3 | 3 | 3 | 3 | 3 | 3 | 3 | 3 | 3 | 3 | 3 |
| Average | 2.6 | 2.6 | 2.9 | 2.8 | 2.1 | 2.6 | 2.8 | 2.5 | 1.9 | 2.6 | 2.8 | 2.3 | 2.2 |  |

## Table S7: Characteristics of Included Studies

| **Program Name, Author, Year, Study Design, location.** | **Total study population (n), Population Type (Adult/Child)** | **Objective** | **Oral Condition** | **Treatment/Intervention description** | **Description of comparator** |
| --- | --- | --- | --- | --- | --- |
| **Quantitative Studies** | | | | | |
| Smiles not Tears , Smith et al. ,2016, Survey, NSW | 61 Aboriginal Health Workers (AHW) from Aboriginal Controlled Community Health Services (ACCHS) | Evaluate AHW oral health training course | Caries | Early childhood oral health education training course | nil |
| Smiles not Tears , Smith et al. ,2018, Quasi Experimental, NSW | 189 Aboriginal children 0-30 months | Decrease caries via AHW led oral health promotion | Caries | Family dental education | Historical dataset |
| Kimberley Dental Team (KDT) , Patel et al. ,2023, Case Report, Kimberleys, WA | 6365 Aboriginal people in the Kimberleys | Process evaluation of KDT | Caries & periodontitis | KDT Program | nil |
| Baby Teeth Talk , Jamieson et al.,2018, Randomised Controlled Trial, SA | 449 Aboriginal mothers and children | Assess if culturally safe dental education decreased caries | Caries | Culturally safe early childhood dental program | Treatment as usual |
| Baby Teeth Talk , Jamieson et al. ,2019, Randomised Controlled Trial, SA | 449 Aboriginal mothers and children | Assess if culturally safe dental education decreased caries if delivered earlier; with 5 yr follow up | Caries | Culturally safe early childhood dental program | Treatment as usual |
| Baby Teeth Talk , Ju et al. ,2023, Randomised Controlled Trial, SA | 449 Aboriginal mothers and children | Assess if culturally safe dental education decreased caries | Caries | Culturally safe early childhood dental program | Treatment as usual |
| Baby Teeth Talk , Smithers et al.,2021, Randomised Controlled Trial, SA | 449 Aboriginal mothers and children | Compare diet intake, anthropometric measures, blood pressure after Baby Teeth Talk intervention | dietary intake | Culturally safe early childhood dental program | Treatment as usual |
| Baby Teeth Talk , Smithers et al.,2017, Randomised Controlled Trial, SA | 449 Aboriginal mothers and children | Compare diet intake, anthropometric measures, blood pressure after Baby Teeth Talk intervention | dietary intake | Culturally safe early childhood dental program | Treatment as usual |
| Strong Teeth for Little Kids , Divaris et al.,2013, Randomised Controlled Trial, Remote NT | 543 Aboriginal Children 2-5YO | Determine effect of fluoride varnish on caries prevention according to tooth anatomy and baseline tooth pathology | Caries | 6 monthly fluoride varnish and oral health promotion for 2 years | dental examination only at baseline and 2 years |
| Strong Teeth for Little Kids , Slade et al.,2011, Community Randomised Trial, Remote NT | 543 Aboriginal Children 2-5YO | Determine effect of fluoride varnish on caries prevention according to tooth anatomy and baseline tooth pathology | Caries | 6 monthly fluoride varnish and oral health promotion for 2 years | dental examination only at baseline and 2 years |
| Strong Teeth for Little Kids , Chondur et al.,2024, Quasi Experimental, Remote NT | 64399 Aboriginal and non-Aboriginal Children in remote NT | Measure changes in caries reduction after community water fluoridation in remote NT | Caries | community water fluoridation | No community water fluoridation |
| Minimal Intervention, Hall Technique, Atraumatic Restorative Technique (MI HT-ART) in Australian Aboriginal Communities , Arrow et al.,2021, Cluster Randomised Trial, Kimberleys, WA | 338 Aboriginal children (<6YO) | Evaluate ART-HT to manage ECC in remote Aboriginal communities | Caries/DMFT | Atraumatic restorative treatment and hall technique | Treatment as usual (e.g. visiting dentists) |
| MI HT-ART in Australian Aboriginal Communities , Arrow et al.,2022, Cluster Randomized Trial, Kimberleys, WA | 338 Aboriginal Children (<6YO) | Measure oral health related quality of life and dental anxiety from ART | Caries | Atraumatic restorative treatment and hall technique | Treatment as usual (e.g. visiting dentists) |
| PerioCardio , Kapellas et al.,2013, Randomised Controlled Trial, NT | 273 Aboriginal and Torres Strait Islander Australian Adults 18<YO | Assess oral health effects of periodontal therapy | Periodontitis | Single visit full mouth non-surgical periodontal therapy | No treatment |
| PerioCardio , Kapellas et al.,2014, Randomised Controlled Trial, NT | 273 Aboriginal and Torres Strait Islander Australian Adults 18<YO | Assess cardiac effects of periodontal therapy | Periodontitis | Single visit full mouth non-surgical periodontal therapy | No treatment |
| No name , Parker & Jamieson,2007, Case Study, Port Augusta, SA | 7560 Aboriginal and non-Aboriginal Children | Compare socio-demographics and oral health between children attending an Aboriginal dental clinic and a Government dental clinic | Caries/DMFT | ACCHS-based Oral Health Services | Existing government dental service |
| No name , Ju et al. ,2017, Randomised Controlled Trial, Port Augusta, SA | 400 Aboriginal and Torres Strait Islander rural-dwelling adults | Determine effect of oral health literacy intervention on oral health literacy related outcomes among rural-dwelling Indigenous Australian adults | All | 5 interactive context specific oral health literacy workshops | No intervention |
| No name , March et al.,2023, Case Study, Dalby, QLD | Unclear Aboriginal people | Describe costs and service code trends in rural Indigenous dental clinic | All | Rural Indigenous dental clinic | N/A |
| No name , Kruger et al.,2010, retrospective process evaluation, WA | Unclear Aboriginal adults and children in rural Western Australia | Compare use of Aboriginal Medical Service based dental clinics and a rural community clinic | N/A | N/A | n/A |
| No Name , Roberts-Thomson et al.,2019, Cluster Randomized Trial, Rural NSW | 384 Aboriginal children in remote communities | Measure effectiveness of silver fluoride compared to atraumatic restorative technique for Aboriginal children in remote communities | Caries and dental infections | silver fluoride | atraumatic restorative technique |
| No Name , Roberts-Thomson et al.,2010, Cluster Randomized Trial, Remote NT | 666 Aboriginal and Torres Strait Islander children in remote communities | Measure impacts and outcomes of oral health promotion for remote Indigenous children | All | oral health promotion (6 monthly fluoride varnish, oral health education, community oral health promotion, training of primary health care staff. | No treatment |
| No Name , Stormon,2022, Retrospective, Rural QLD | 525 Aboriginal and Torres Strait Islander school-going children | Process evaluation of school based dental service's consent process | N/A | N/A | N/A |
| No Name , Johnson et al.,2014, Longitudinal, Northern Peninsula Area, Queensland | 324 Aboriginal and Torres Strait Islander school children | Identify changes in DMFT in population after community water fluoridation introduced | Caries/DMFT | Community water fluoridation | N/A |
| No Name , Dimitropoulos et al.,2020, Case Study, Central Northern NSW | 104 Aboriginal Children (5-17YOs) | Process evaluation of school fluoride varnish program | Caries | School based Fluoride Varnish Program | N/A |
| No Name , Dimitropoulos et al.,2018, Cross-Sectional, Rural NSW | 163 Aboriginal children, parents, school staff and community health workers | Assess community oral health needs | All | N/A | N/A |
| No Name , Dimitropoulos et al.,2020, Cohort Study, Rural NSW | 88 Aboriginal children | Measure changes in caries, oral hygiene, gingivitis, and parent perceptions | Caries and gingivitis | Oral hygiene program | N/A |
| No Name , Lalloo et al.,2015, Retrospective cohort study, WA, NT, SA, ACT, QLD, Tas | 97809 Aboriginal and Torres Strait Islander and non-Aboriginal and Torres Strait Islander children | Assess if water fluoridation closes the gap in dental caries between Indigenous and non-Indigenous children | Caries | Water Fluoridation | Non-Water Fluoridated area |
| No Name , Gwynne et al.,2017, Case Study, Rural NSW | Unclear Aboriginal people in NSW | Compare productivity of two dental models | All | Fly-in fly-out dental team (Model A) | ACCHS-led dental team (Model B) |
| No name , Irving et al.,2017, Cross-Sectional, Rural NSW | 49 Aboriginal children and parents | Examine views of children and parents accessing an Aboriginal dental health service | All | Aboriginal dental health service | N/A |
| No Name, Lalloo et al.,2021, Quasi Experimental, Northern Peninsula Area, Queensland | 408 Aboriginal and Torres Strait Islander children residing in a remote Australian community | Assess caries reduction of "Big Bang" program compared to treatment as usual | Caries | Preventive dental exam with treatment (including fissure sealants, povidone-iodine, and fluoride varnish) | Problem based dental visiting pattern |
| No Name, Tadakamadla et al.,2020, Quasi Experimental, Northern Peninsula Area, Queensland | 408 Schoolchildren in a remote Aboriginal community (5-17YO) | Measure surface-specific caries preventative effect of preventative interventions in a remote Indigenous community | Caries | Fissure sealant, povidone-iodine, and fluoride varnish | Usual care (e.g. emergency treatment and restorations) |
| No Name, Kroon et al.,2019, Longitudinal, Northern Peninsula Area, Queensland | 2434 Aboriginal Children (4-15 YO) | Identify trends in DMFT | Caries/DMFT | community water fluoridation, dental treatment | N/A |
| **Qualitative studies** | | | | | |
| KDT,Patel et al. ,2021, Qualitative, Kimberleys, WA | 80,Aboriginal adults living in East Kimberley | Explore perceptions and attitudes towards dental services among Aboriginal adults in remote Kimberley communities | N/A | Dental services in remote Kimberley communities | N/A |
| MI HT-ART in Australian Aboriginal Communities, Piggott et al.,2021, Qualitative ,Kimberleys, WA | 29,Parents and carers of children | Evaluate ART-HT to manage ECC in remote Aboriginal communities | Caries/DMFT | Atraumatic restorative treatment and hall technique | Treatment as usual (e.g. visiting dentists) |
| Pika Wiya,Parker et al.,2005, Case Study ,Port Augusta, SA | Unclear,Aboriginal children and adults | Planning and implementing oral health program | N/A | ACCHS-based Oral Health Services | Existing government and private dental services |
| Pika Wiya,Jamieson, Parker & Richards,2008, Qualitative,Port Augusta, SA | 34,Rural-dwelling Aboriginal and Torres Strait Islander Australians | Explore perceptions of oral health | N/A | N/A | N/A |
| Goondir,Mangoyana et al.,2022, Qualitative,Dalby, QLD | 38,Aboriginal community Elders, community health support group members and management and staff | Describe community opinions of student dental clinic within rural ACCHS | All | Student dental clinic in ACCHS | N/A |
| AKction,Kelly et al.,2024, Case Study,SA | Unclear,First Nations people experiencing kidney disease | Codesign culturally safe oral health care | Caries & periodontitis | Oral health promotion and examination | nil |
| Grinnin Up Mums and Bubs,Kong et al.,2020, Qualitative,NSW | 14,Aboriginal health staff involved in antenatal care | Explore perspectives of Aboriginal health staff on maternal oral health | N/A | N/A | N/A |
| Grinnin Up Mums and Bubs,Kong et al.,2021, Qualitative,NSW | 12,Aboriginal pregnant women | Explore perspectives of Aboriginal pregnant women on oral health | N/A | N/A | n/A |
| No Name,Dimitropoulos et al.,2019, Qualitative,Rural NSW | 15,Oral Health Aides in Rural NSW | Thematic analysis of school staff perspectives on in-school toothbrushing program in Aboriginal communities | All | In-school toothbrushing program | N/A |
| No name,Gwynne et al.,2016, Case Study,Rural NSW | Unclear,Aboriginal people in remote NSW | Describe co-design of Aboriginal rural dental program | All | Aboriginal co-designed dental health program | N/A |
| No Name,Walker et al.,2022, Qualitative,Rural QLD | 27,Rural Aboriginal people in Queensland | Explore rural Aboriginal community strategies to inform delivery of oral healthcare | All | N/A | N/A |
| **Mixed Methods** |  |  |  |  |  |
| Smiles not Tears,Smith et al. ,2020,Mixed Methods,NSW | 110,Parents of Aboriginal children 0-30 months, AHW and managers from ACCHSs | Evaluate oral health education program | Caries | AHW led oral health education | nil |
| KDT,Patel et al.,2023,Mixed Methods,Kimberleys, WA | N/A,Aboriginal people in the Kimberleys | Report KDT activities | Caries & periodontitis | KDT Program | nil |
| Strong Teeth,Spencer et al.,2010,Mixed Methods,Remote NT | N/A,Remote Aboriginal communities | Describe rationale and feasibility of fluoridating remote Indigenous communities | Caries | community water fluoridation | No community water fluoridation |
| Pika Wiya,Parker et al.,2012,Mixed Methods,Port Augusta, SA | 1329,Aboriginal children | Describe planning, implementation and evaluation of ACCHS-based dental clinic | N/A | N/A | N/A |
| Grinnin Up Mums and Bubs,Kong et al.,2021,Mixed Methods,NSW | 7,Aboriginal Health Workers | Pilot test oral health training for Aboriginal Health Workers | N/A | N/A | N/A |
| No Name,Skinner et al.,2020,Mixed Methods,Rural NSW | 536,Aboriginal children attending schools, and Aboriginal dental assistants | Evaluation of Aboriginal dental assistant application of fluoride varnish | Caries | Aboriginal dental assistant apply fluoride varnish | N/A |
| No Name,Campbell et al.,2015,Mixed Methods,NSW | Unclear,ACCHS Staff | Explore experiences of ACCHSs in oral health care | N/A | N/A | N/A |
| Baby Teeth Talk,Hammersley et al.,2022,Case Study,SA | 449,Aboriginal mothers and children | Report strategies improving participant engagement | Caries | Culturally safe early childhood dental program | Treatment as usual |
| Big Bang,Kularatna et al.,2020,Economic,Northern Peninsula Area, Queensland | 408,Children 5-18 YO (>95% were Indigenous) | Assess cost effectiveness and cost utility of "Big Bang" program compared to treatment as usual | Caries & gingivitis | Preventive dental exam with treatment (including fissure sealants, povidone-iodine, and fluoride varnish) | Problem based dental visiting pattern |
| NT Community Water Fluoridation,Zhao et al.,2023,Economic,Remote NT | Unclear,Remote Aboriginal communities in the NT | Measure cost effectiveness of community water fluoridation in remote NT communities | Caries | Community water fluoridation | No community water fluoridation |
| NT Community Water Fluoridation,Ehsani & Bailie,2007,Economic,Remote NT | N/A,N/A | Measure costs of water fluoridation in remote NT communities and describe challenges | N/A | community water fluoridation | N/A |

## Table S8: Included Studies Assessed with Lowitja Criteria

|  | **Lowitja Criteria** | | | | | | | | | |
| --- | --- | --- | --- | --- | --- | --- | --- | --- | --- | --- |
| **Author & Year** | **Shared Responsibility and Partnerships** | **Engagement** | **Capacity building** | **Equity** | **Accountability** | **Evidence-Based** | **Holistic Concept of Health** | **Cultural Competence** | **Data Governance and Intellectual Property** | **Capitalising on Indigenous Strengths** |
| Smith et al., 2016 | AHW feedback | AHW feedback | AHW feedback and training | Increase dental education for Aboriginal families | AHW feedback | AHW feedback | AHW led to decrease barriers to access | AHW led | Education to be disseminated in community | AHW feedback |
| Smith et al., 2018 | AHW informed | AHW led | AHW training | Increase dental education for Aboriginal families | Evaluation performed | Evidence-based intervention | AHW led to decrease barriers to access | AHW led | Unclear | AHW led |
| Smith et al., 2020 | Feedback from Aboriginal parents, AHW and ACCHS managers | Feedback from Aboriginal parents, AHW and ACCHS managers | Feedback from Aboriginal parents, AHW and ACCHS managers | Increase dental education for Aboriginal families | Feedback from Aboriginal parents, AHW and ACCHS managers | Evidence-based intervention | AHW led to decrease barriers to access | AHW led | Unclear | AHW led |
| Patel et al., 2015 | Unclear | Unclear | Unclear | Aim to identify barriers for dental workforce in Kimberley’s | Unclear | Evidence based intervention | Unclear | Unclear | Unclear | Unclear |
| Patel et al., 2023 | Partner with Aboriginal Medical Services (AMS), Aboriginal governance group | Partner with AMS, Aboriginal governance group | Employment of Aboriginal oral health promotion officer, partner with AMS | Improve dental care access for Aboriginal people in the Kimberleys (rural) | Evaluations performed, community feedback | Evidence based intervention | Incorporation of community and societal determinants in program logic | Partner with AMS, Aboriginal governance group, and Aboriginal oral health promotion officer | Unclear | Partner with AMS, Aboriginal governance group, and Aboriginal oral health promotion officer |
| Patel et al., 2023 | Partner with AMS, Aboriginal governance group | Partner with AMS, Aboriginal governance group | Employment of Aboriginal oral health promotion officer, partner with AMS | Improve dental care access for Aboriginal people in the Kimberleys (rural) | Evaluations performed, community feedback | Evidence based intervention | Consideration of community and societal determinants in program activities | Partner with AMS, Aboriginal governance group, and Aboriginal oral health promotion officer | Unclear | Partner with AMS, Aboriginal governance group, and Aboriginal oral health promotion officer |
| Patel et al. , 2021 | Partnering with community for their feedback | Partnering with community for their feedback | Aboriginal liaison officers involved in interview development, data collection, | Aim to increase profile of rural Aboriginal people's dental service experiences | Evaluation of current oral health services | Evidence based evaluation plan | Discussions of social determinants and impacts of dental health on general wellbeing |  | Final manuscript reviewed by Aboriginal leader of East Kimberley | Yarning methodology, purposive sampling via Aboriginal liaison officer utilising existing networks |
| Jamieson et al., 2018 | Aboriginal focus groups and reference groups | Aboriginal focus groups and reference groups | Unclear | Increase dental education for Aboriginal families | Unclear | Evidence based intervention | Consideration of family and community | Aboriginal focus groups and reference group | Unclear | Aboriginal focus groups and reference group |
| Jamieson et al. , 2019 | Aboriginal focus groups and reference group | Aboriginal focus groups and reference group | Unclear | Increase dental education for Aboriginal families | Unclear | Evidence based intervention | Consideration of family and community | Aboriginal focus groups and reference group | Unclear | Aboriginal focus groups and reference group |
| Ju et al. , 2023 | Aboriginal focus groups and reference group | Aboriginal focus groups and reference group | Unclear | Increase dental education for Aboriginal families | Unclear | Evidence based intervention | Consideration of family and community | Aboriginal focus groups and reference group | Unclear | Aboriginal focus groups and reference group |
| Hammersley et al., 2022 | Aboriginal reference group with Indigenous leaders in health | Aboriginal reference group with Indigenous leaders in health | Aboriginal reference group with Indigenous leaders in health; authors note this could be improved by employing Aboriginal Health Workers who were based in ACCHOs | cultural competence training and refreshers for all staff | Newsletters and presentations to ACCHOs | Evidence based intervention | Considered community, spiritual, emotional, physical and environmental health | Study design accounting for difficulty reaching participants | Dissemination via presentations at hospitals and meeting and provision of resources with heatlh professionals | Snowball recruitment, recruitment in social groups |
| Smithers et al., 2021 | Unclear | Unclear | Unclear | Increase dental education for Aboriginal families | Unclear | Evidence based intervention | Unclear | Unclear | Unclear | Unclear |
| Smithers et al., 2017 | Aboriginal reference group | Active community and family engagement via Aboriginal research officer | Unclear | Collaborative process | Unclear | Evidence based intervention | Inclusion of oral health intervention as part of wider diet changes, acknowledgement of social, family,community, country and spiritual health factors | Aboriginal research officer heavily involved in family liaison, Aboriginal focus groups and reference group | Aboriginal research officer presented to community groups and at Indigenous culture celebrations | Aboriginal research officer heavily involved in family liaison, Aboriginal focus groups and reference group |
| Kularatna et al., 2020 | Unclear | Community liaisons employed | Community liaisons employed | Improve dental access for rural/remote people | Economic evaluation performed | Evidence based intervention and evaluation of cost effectiveness performed | Quality of Life measured | Unclear | Present results to community | Unclear |
| Lalloo et al., 2021 | Community consultation | Community consultation | Unclear | Improve dental access for rural/remote people | Clinical effectiveness evaluated | Evidence based intervention and evaluation of clinical effect performed | Unclear | Unclear | Unclear | Unclear |
| Tadakamadla et al., 2020 | Unclear | Unclear | Unclear | Improve dental access for rural/remote people | Clinical effectiveness evaluated | Evidence based intervention and evaluation of clinical effect performed | Unclear | Unclear | Unclear | Unclear |
| Kroon et al., 2019 | Unclear | Unclear | Unclear | Unclear | Unclear | Evidence based intervention | Unclear | Unclear | Unclear | Unclear |
| Divaris et al., 2013 | Unclear | Unclear | Unclear | Unclear | Unclear | Evidence based intervention | Unclear | Unclear | Unclear | Unclear |
| Slade et al., 2011 | Indigenous Reference Group | Indigenous Reference Group | Training of remote health staff and AHWs for oral health promotion and dental service referrals | Increase access for remote people | Unclear | Evidence based intervention | Not measured | Unclear | Unclear | Indigenous Reference Group |
| Spencer et al., 2010 | Communities requested water fluoridation | Communities requested water fluoridation | Attempted but unsuccessful | Aim to improve dental health of remote Indigenous peoples | Unsuccessful, fluoridation plant could not be community-owned | Evidence based intervention | Response to environmental and social determinants of oral health | Unclear | Unclear | Unclear |
| Zhao et al., 2023 | Unclear | unclear | Unclear | Aim to improve oral health of remote Aboriginal communities | Economic evaluation performed | Economic evaluation performed; evidence-based intervention | Social and environmental factors aimed to be addressed via community water fluoridation | Unclear | Unclear | Unclear |
| Chondur et al., 2024 | Unclear | Unclear | Unclear | Aim to improve oral health of remote Aboriginal communities | Clinical effectiveness evaluated | Evidence based intervention | Unclear | Unclear | Unclear | None |
| Ehsani & Bailie, 2007 | Unclear | Unclear | Unclear | Aim to improve fluoride exposure for remote Aboriginal communities | Costs and challenged evaluated | Evidence based intervention and evaluation | Unclear | Unclear | Unclear | Unclear |
| Arrow et al., 2021 | Aboriginal Reference Group with members from ACCHOs | Aboriginal Reference Group and Aboriginal Research Assistant | Employment of Aboriginal Research Officer | Improved dental outcomes for Aboriginal people in remote areas | Clinical effectiveness evaluated | Evidence based intervention and evaluation | Unclear | Aboriginal research assistant guided activities, feedback sought from participants | Unclear | Aboriginal research assistant, Aboriginal reference groups and community meetings informed program development. |
| Piggott et al., 2021 | Unclear | Feedback sought about program | Unclear | Improved dental outcomes for Aboriginal people in remote areas | Feedback sought about program | Evidence based intervention | Family, community engagement assessed as well as satisfaction with dental care | Interviews led by Aboriginal Research Officer, Kriol language interpreting | Unclear | Yarning methodology |
| Arrow et al., 2022 | Aboriginal Reference Group with members from ACCHOs | Aboriginal Reference Group with members from ACCHOs | Employment of Aboriginal Research Officer | Improved dental outcomes for Aboriginal people in remote areas | Quality of life and dental anxiety outcomes evaluated | Evidence based intervention and evaluation | Holistic view of dental health, inclusion of dental anxiety and quality of life | Aboriginal research officer collected data from participants | Unclear | Aboriginal research officer, Aboriginal reference groups and community meetings informed program development. |
| Kapellas et al., 2013 | Unclear | Unclear | Employment of local Aboriginal Health Workers to liaise with participants | Improvement to dental care access for participants in remote areas and living in correctional services | Unclear | Evidence based intervention | Unclear | Employment of local Aboriginal Health Workers to liaise with participants | Unclear | Unclear |
| Kapellas et al., 2014 | Unclear | Unclear | Unclear | Improvement to dental care access for participants in remote areas and living in correctional services | Unclear | Evidence based intervention | Unclear | Unclear | Unclear | Unclear |
| Parker et al., 2005 | Working group with local ACCHS representation | Working group with local ACCHS representation | Support of dental clinic within ACCHS infrastructure, engagement with AHW | Improve dental service access for rural Aboriginal people | Program evaluated | Findings contribute to program development | Unclear | Working group with local ACCHS representation, engagement of AHW | Unclear | ACCHS led program development |
| Parker & Jamieson, 2007 | ACCHS-led and funded program | ACCHS-led | Unclear | Improve dental service access for rural Aboriginal people | Program evaluated | Findings contribute to program development | Unclear | ACCHS-led | Unclear | Unclear |
| Jamieson, Parker & Richards, 2008 | Unclear | Unclear | Unclear | Improve dental service/health promotion access for rural Aboriginal people | Evaluating social, cultural and environmental context of oral health | Evaluating social, cultural and environmental context of oral health | Evaluating social, cultural and environmental context of oral health | Unclear | Unclear | Unclear |
| Parker et al., 2012 | Steering committee with Aboriginal representation, feedback sought from Aboriginal staff | Steering committee with Aboriginal representation, feedback sought from Aboriginal staff | Working with Aboriginal Health Worker | Improve access and cultural safety by being based in ACCHS | Regular informal evaluation, and major attendance evaluation | Evaluated via regular feedback, findings used for program development | Health promotion in schools | Aim of increasing cultural safety by being based in ACCHS | Unclear | Engagement of Aboriginal Health Worker |
| Ju et al. , 2017 | Pilot testing with community | Pilot testing with community | Indigenous project staff employed | Aimed to improve oral health outcomes for rural Aboriginal people | Development of instrument to measure oral health literacy with community | Evaluated program | Evaluation of clinical (e.g. oral health literacy) and psycho-social outcomes e.g. self-efficacy, sense of control | Indigenous project officers recruited for participant retention | Unclear | Community networks used for recruitment, Indigenous project officers collected data and managed recruitment and retention |
| March et al., 2023 | ACCHS-based clinic | Unclear | Unclear | Improve access and cultural safety by being based in ACCHS | Unclear | Unclear | Unclear | Unclear | Unclear | Unclear |
| Mangoyana et al., 2022 | Consultation with Aboriginal and Torres Strait Islander Community Controlled Health Services (ATSICCHS) to develop evaluation plan | Consultation with Aboriginal and Torres Strait Islander Community Controlled Health Services (ATSICCHS) to develop evaluation plan | Student clinic within ACCHS | Improve access and cultural safety of dental care for rural Indigenous peoples; free transport and free treatment | Qualitative evaluation of new dental service | Evidence based program and evaluation plan | Qualitative analysis of cultural safety, wellbeing and oral health literacy | ACCHS advised on focus group methods, community representatives present at focus groups, community cross-checking of manuscript draft by ACCHS representatives | Community cross-checking of manuscript draft by ACCHS representatives | Yarning methodology |
| Kelly et al., 2024 | Aboriginal reference group | Aboriginal reference group | Inclusion of Kidney Warriors in research team | Collaborative process | Collaborative feedback at all stages | Evidence base for intervention unclear | Understanding of social determinants of health, oral health as part of overall wellbeing for people with kidney disease | Incorporation of cultural practices | Program still continuing; unclear | Indigenous methodologies: Dadirri, Ganma and Clinical yarning |
| Kong et al., 2020 | Co-design with Aboriginal antenatal care providers | Co-design with Aboriginal antenatal care providers | Findings to be used to co-design dental program with Aboriginal antenatal care providers | Aim to improve oral health access for pregnant Aboriginal women | Evaluation of current models of care | Evidence based methodology | Discussions of social and historical determinants, trust, and continuity of care | Yarning methodology used, Aboriginal people respected as owners of knowledge | Aboriginal people respected as owners of knowledge, findings to lead to co-design of dental health program; knowledge gained will be taught/shared to Aboriginal Health Workers | Yarning methodology |
| Kong et al., 2021 | Co-design project with Aboriginal antenatal care providers | Co-design with Aboriginal antenatal care providers | Findings to be used to co-design dental program with Aboriginal antenatal care providers, core research team included Aboriginal researchers | Aim to improve oral health access for pregnant Aboriginal women | Evaluation of current models of care | Evidence based methodology | Discussions of social and historical determinants, trust, and continuity of care | Yarning methodology used, Aboriginal researchers informed study design, data collection and analysis | Training and knowledge will be taught to Aboriginal Health Workers | Yarning methodology, Aboriginal researchers, co-design with Aboriginal antenatal care providers |
| Kong et al., 2021 | Co-design project with Aboriginal antenatal care providers and Aboriginal researchers, Aboriginal Action Group consulted, feedback from Aboriginal pregnant women and mothers sought. | Co-design project with Aboriginal antenatal care providers, Aboriginal Action Group consulted, feedback from Aboriginal pregnant women and mothers sought. | Training delivered to Aboriginal antenatal care providers, Aboriginal graphic designer employed | Aim to improve oral health access for pregnant Aboriginal women | Evaluation of new program | Evidence based methodology and evaluation | Program acknowledged social, historical and cultural determinants of health/dental health | AHW feedback confirmed cultural competence of program | Training and knowledge taught to Aboriginal Health Workers | Yarning methodology, Aboriginal researchers, co-design with Aboriginal antenatal care providers, Aboriginal graphic designer employed to make artwork for resources |
| Kruger et al., 2010 | Unclear | Unclear | dental clinics based in AMSs | Describe use of services of rural Aboriginal peoples | Process evaluation of rural Aboriginal dental clinics | Evidence based evaluation plan | Unclear | Unclear | Unclear | Unclear |
| Roberts-Thomson et al., 2019 | Unclear | Unclear | Unclear | Increase acceptable dental treatments for remote Aboriginal children | Evaluation of intervention | Evidence based intervention and evaluation plan | Unclear | Unclear | Unclear | Unclear |
| Roberts-Thomson et al., 2010 | Indigenous Reference Group | Indigenous Reference Group, local council oral health promotion training | Indigenous research staff members (with one member from the community), training of primary care staff to provide comprehensive oral health promotion, local community oral health training for community leaders and councils | Aim to improve dental health of rural Indigenous peoples | Evaluation of program | Evidence based intervention and evaluation plan | unclear | unclear | Unclear | Unclear |
| Skinner et al., 2020 | Invitation of ACCHS to participate, work with ACCHS's own dental assistants, | Invitation of ACCHS to participate, work with ACCHS's own dental assistants, | Aboriginal dental assistants received training to provide fluoride varnish | Increase clinical fluoride exposure for rural/remote Aboriginal children | Process evaluation of fluoride varnish program | Evidence based intervention and evaluation plan | unclear | Recognition of ACCHS as leaders of Aboriginal health; Aboriginal dental assistant led intervention | Unclear | Unclear |
| Stormon, 2022 | Unclear | Unclear | Unclear | Aim to highlight service gap for rural Indigenous children in accessing school dental services | Process evaluation of consent process | Evidence based evaluation plan | Unclear | Unclear | Unclear | Unclear |
| Johnson et al., 2014 | Consultation with Elders of the NPA community | Consultation with Elders of the NPA community | Unclear | Improve water fluoridation exposure for remote Indigenous peoples | Evaluation of fluoridation effectiveness | Evidence based intervention and evaluation | unclear | Unclear | Unclear | Unclear |
| Dimitropoulos et al., 2020 | Response to Elders' concerns, community engagement and co-design | Co-design with Elders, representatives from school, Aboriginal Land Council, and Community Health Centres; discussions with Aboriginal Health and Medical Research Council | Engagement of Aboriginal Education Officer to liaise with families | Improved outcomes for Aboriginal people in remote areas | Community leader endorsement, prioritisation; process evaluation | Evidence based intervention | Unclear | Yarn-up methodology | Unclear | Yarn-up methodology |
| Dimitropoulos et al., 2017 | Consultation with ACCHSs | Consultation with ACCHSs | Leading to development of ACCHS based oral health service | Aim to improve ACCHS oral health service delivery | Program evaluation plan developed including feedback and clinical effectiveness | Evidence based evaluation plan | Recognition of ACCHS as leaders of Aboriginal health | Recognition of ACCHS as leaders of Aboriginal health | Knowledge owned and used by ACCHSs | Recognition of ACCHS as leaders of Aboriginal health |
| Dimitropoulos et al., 2020 | Co-design with Aboriginal community | Co-design with Aboriginal community, schools chosen by Elders | Employment and training for Aboriginal dental assistants and Aboriginal health aides | Improved dental outcomes for Aboriginal people in remote areas | Program evaluation including feedback and clinical effectiveness | Evidence based intervention and evaluation plan | Promotion of preventative oral health practices, aim to improve dental health literacy | Aboriginal community co-design and employment of Aboriginal staff | Aboriginal co-design and ownership of program | Aboriginal community co-design and employment of Aboriginal staff |
| Dimitropoulos et al., 2019 | Collaboration with local Aboriginal community | Unclear | Unclear | Improved dental outcomes for Aboriginal people in remote areas | School staff feedback for program evaluation | Evidence based intervention and evaluation plan | Promotion of preventative oral health practices, aim to improve dental health literacy | Collaboration with local Aboriginal community | Unclear | Unclear |
| Lalloo et al., 2015 | Unclear | Unclear | Unclear | Unclear | Unclear | Evidence based intervention | Unclear | Unclear | Unclear | Unclear |
| Gwynne et al., 2017 | Model A - some Aboriginal staff; Model B - ACCHS-led, Aboriginal clinicians | Model A - some Aboriginal staff; Model B - ACCHS-led, Aboriginal clinicians | Model A - some Aboriginal staff; Model B - ACCHS-led, Aboriginal clinicians | Improve dental access for rural/remote people | Program evaluated for productivity | Program evaluated for productivity | Unclear | Model A & B | Unclear | Unclear |
| Gwynne et al., 2016 | steering committee with Aboriginal research group, community elders, ACCHS, and other stakeholders | Priority for local elders and community; steering committee | Dental business owned by community; local people studied Cert III and IV in Dental Assisting and Oral health Promotion supported by scholarships from research group. | improve dental service access for rural Aboriginal people, continual collaboration | Shared measurement to form evaluation plan | Evidence based system of "collective impact methodology" | Recognition of ACCHS as leaders of Aboriginal health | Recognition of ACCHS as leaders of Aboriginal health | Quantitative data (e.g. patient records, bookings) held by ACCHS | Recognition of ACCHS as leaders of Aboriginal health; Aboriginal input and local co-design to create program |
| Irving et al., 2017 | collaborative design with local community, local health service providers, and Aboriginal research group | Priority for local elders and community; collaborative design | Hosted in local AMS, community centre, school, or mobile dental van | improve dental service access for rural Aboriginal people, continual collaboration | Evaluation of child and parent perceptions of dental service | Evidence based intervention and evaluation | Inclusion of parent perceptions in child health (familial/generational approach) | Clinicians trained in Aboriginal culture | Unclear | Collaborative design with Aboriginal peoples |
| Walker et al., 2022 | Requested by community, Indigenous research team were community members, local Indigenous research assistant employed, local Health Action Group formed | Requested by community, Indigenous research team were community members, local Indigenous research assistant employed | Employment of local Indigenous research assistant for participant recruitment and data collection | Aim to improve dental health of rural Indigenous peoples | Qualitative evaluation of current oral health services | Qualitative evaluation to be utilised by oral health service planners | Social and environmental factors discussed e.g. financial barriers, transport, cultural safety, oral health promotion | Data analysis checked by local Health Action Group, Indigenous research assistant informed data collection | Unclear | Purposive and snowball recruitment led by Indigenous peoples |
| Campbell et al., 2015 | Aboriginal Health & Medical Research Council (AH&MRC)-led project | AH&MRC led project, engaged ACCHS perspectives | Recommendation for stable funding for oral health services in ACCHSs | Aim to improve ACCHS oral health service delivery | Evaluation of current ACCHS oral health service delivery | Evaluation of current ACCHS oral health service delivery | Recognition of ACCHS as leaders of Aboriginal health | Aboriginal led research project | Aboriginal led research with knowledge gained recommending stable funding | Aboriginal led research project, capitalising on ACCHS knowledge |

# Supplementary Figures

## Figure S1: Histogram of Average QuADS Score and Number of Studies


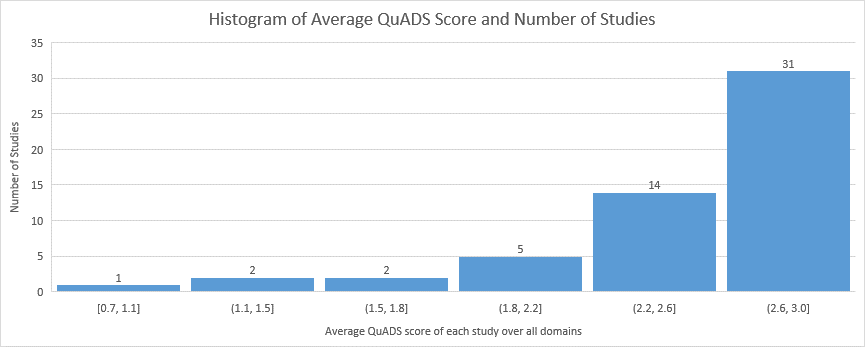


References

1. Page MJ, McKenzie JE, Bossuyt PM, et al. The PRISMA 2020 statement: an updated guideline for reporting systematic reviews. *bmj.* 2021;372.

2. Cooke A, Smith D, Booth A. Beyond PICO: the SPIDER tool for qualitative evidence synthesis. *Qualitative health research.* 2012;22(10):1435-1443.
